# Aging-dependent microglial heterogeneity worsens outcomes in models of traumatic brain injury

**DOI:** 10.1172/JCI196112

**Published:** 2026-04-02

**Authors:** Zhichao Lu, Yi Shuai, Chenxing Wang, Zongheng Liu, Ziheng Wang, Qianqian Liu, Rui Jiang, Jue Zhu, Yongqi Zhu, Weiquan Liao, Xingjia Zhu, Jingwei Zhao, Kaibin Shi, Wei Shi, Peipei Gong

**Affiliations:** 1 Department of Neurosurgery, Research Center of Clinical Medicine, Affiliated Hospital of Nantong University, Medical School of Nantong University, Nantong, China.; 2 Neuro-Microscopy and Minimally Invasive Translational Medicine Innovation Center, Affiliated Hospital of Nantong University, Nantong, China.; 3 Faculty of Medicine, The Chinese University of Hong Kong, Shatin, Hong Kong Special Administrative Region, China.; 4 Department of Neurosurgery, Zhejiang Provincial Hospital of Chinese Medicine, The First Affiliated Hospital of Zhejiang Chinese Medical University, Hangzhou, China.; 5 MOE Frontier Science Centre for Precision Oncology, University of Macau, Macau Special Administrative Region, China.; 6 School of Public Health and Preventive Medicine, Monash University, Melbourne, Victoria, Australia.; 7 Department of General Surgery, Xinhua Hospital Affiliated to Shanghai Jiao Tong University School of Medicine, Shanghai, China.; 8 Department of Neurology, China National Clinical Research Center for Neurological Diseases, Beijing Tiantan Hospital, Capital Medical University, Beijing, China.; 9 Chinese Institute for Immunology, Chinese Institutes for Medical Research, Beijing, China.

**Keywords:** Immunology, Inflammation, Neuroscience, Drug therapy, Innate immunity, Neurodegeneration

## Abstract

Traumatic brain injury (TBI) disproportionately affects the elderly, yet the underlying mechanisms remain unclear. Here, we demonstrate that aged TBI brains predominantly harbor proinflammatory NLRP3^+^ microglia, in stark contrast to the neuroprotective Lysozyme^+^ microglia prevalent in young TBI brains. This age-dependent microglial dichotomy correlates with elevated mortality and impaired recovery in aged TBI mice. By leveraging an integrative multiomics approach combined with metabolomics and epigenome analysis, we identified a previously unrecognized link between enhanced glycolysis and the proinflammatory chromatin landscape in NLRP3^+^ microglia. Further investigation identified ELF1 as a key transcription factor driving NLRP3^+^ microglia formation. Importantly, ablation of ELF1 reversed age-associated microglial dysfunction and improved TBI outcomes. Finally, we report that Imeglimin, a clinically approved antihyperglycemic agent capable of crossing the blood-brain barrier, inhibits ELF1 and reverses microglial phenotype, reducing acute mortality rate and leading to improved functional recovery of aged mice with TBI. Our work elucidates the mechanistic basis of age-dependent TBI outcomes, reveals the crosstalk between metabolic rewiring and epigenetic regulation in microglial aging, and identifies ELF1 as a promising therapeutic target for improving TBI outcomes.

## Abstract 2

Aging-related inflammatory microglia worsen outcomes of traumatic brain injury

## Graphical abstract

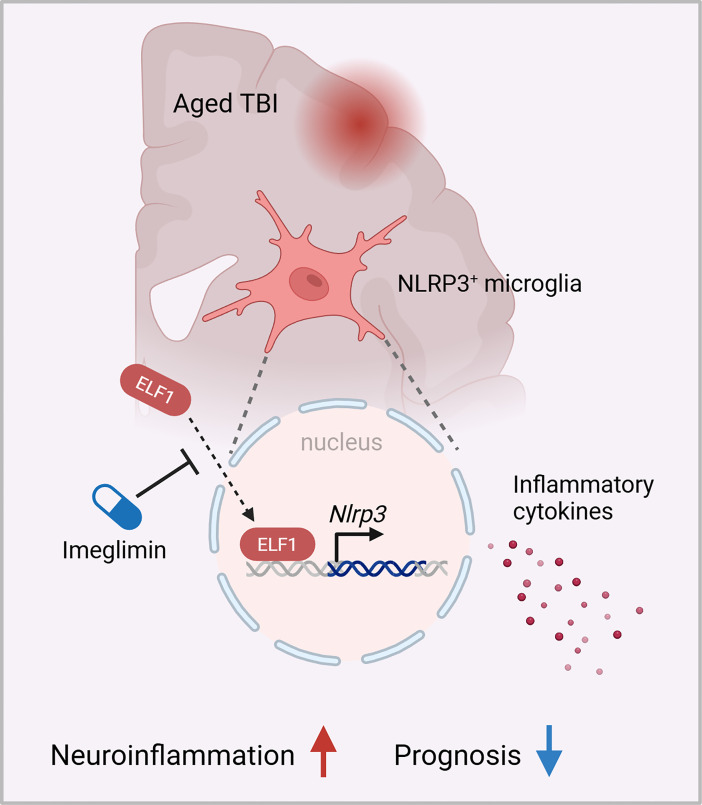

## Introduction

Traumatic brain injury (TBI) is a leading cause of death and long-term disability globally (1, 2), but effective pharmacologic treatments are lacking (3). Epidemiologically, the frequency of hospital admissions is highest in patients over 65 years old, with a mortality rate more than double that of younger populations. Additionally, aged survivors often experience substantial impairments in quality of life (4, 5). Aging has profound effects on the immune responses, but how these age-related changes worsen the prognosis of TBI remains unclear (6, 7).

Microglia are resident immune cells in the CNS and play a key role in maintaining CNS homeostasis and mediating responses to injury (8, 9). In aging and neurodegenerative conditions, microglia lose their balanced functional profile, exhibiting impairments such as enhanced proinflammatory cytokine production, increased ROS release, and compromised phagocytosis marked by lysosomal deposition (10–12). Recent single-cell transcriptomics studies have revealed remarkable heterogeneity among microglia, including disease-associated microglia (DAMs), which is a dysfunctional microglia phenotype (13–15). While these studies highlight microglial plasticity, critical gaps remain in our understanding of how aging reshapes the composition and functional properties of microglial subpopulations after TBI. Specifically, it remains unclear whether age-driven shifts in microglial subsets contribute to the worse outcomes observed in aged patients with TBI and, if so, what molecular and cellular clues underly these effects.

Emerging evidence suggests that metabolic rewiring and epigenetic regulation are key drivers of microglial functional polarization (16, 17). For instance, proinflammatory microglia often rely on glycolysis for energy, whereas antiinflammatory or homeostatic microglia favor oxidative phosphorylation (OXPHOS) and the TCA cycle (17). Aging has been shown to disrupt microglial metabolism, but how these metabolic changes intersect with transcriptional and epigenetic alterations to shape microglial heterogeneity after TBI is not well defined. Additionally, transcription factors (TFs) that orchestrate age-specific microglial phenotypes remain largely uncharacterized, presenting a critical gap in identifying potential therapeutic targets.

Given the unknown reasons for age-dependent TBI outcomes and the critical role of microglia in neuroinflammation, we hypothesized that aging induces selective expansion of proinflammatory microglial subsets and impairment of neuroprotective subsets, driven by coordinated metabolic reprogramming and epigenetic remodeling. To test this hypothesis, we examined the injured cerebral hemisphere in aged and young mice with TBI by single-cell sequencing to investigate the effects of aging on the brain immune microenvironment after TBI. After TBI, the brains of aged patients and mice have a stronger inflammatory response, as evidenced by a massive infiltration of immune cells characterized by a strong microglia response. In this study, we identified a population of NLRP3^+^ microglia that worsened the prognosis of aged patients and mice after TBI. The results of untargeted metabolomics and ATAC-seq (assay for transposase-accessible chromatin using sequencing) identified impaired energy metabolism in NLRP3^+^ microglia with higher chromatin accessibility to proinflammatory markers. Using the SCENIC algorithm and motif enrichment, combined with the CRISPR/Cas9 system, we identified TFs (*ELF1*, *NFKB2*, *RELA*, *FLI1*, *NFKB1*, *JUN*, *JUNB*, and *FOSB*) that may collectively regulate NLRP3^+^ microglia formation, with ELF1 playing the most prominent role. Imeglimin, a newly marketed antihyperglycemic agent (18), blocks the action of ELF1 and reduces the population of NLRP3^+^ microglia after TBI, leading to an improved prognosis in mice, especially aged mice, after TBI.

## Results

### Microglia responses are more severe in aged TBI patients and mice.

**Figure 1:**
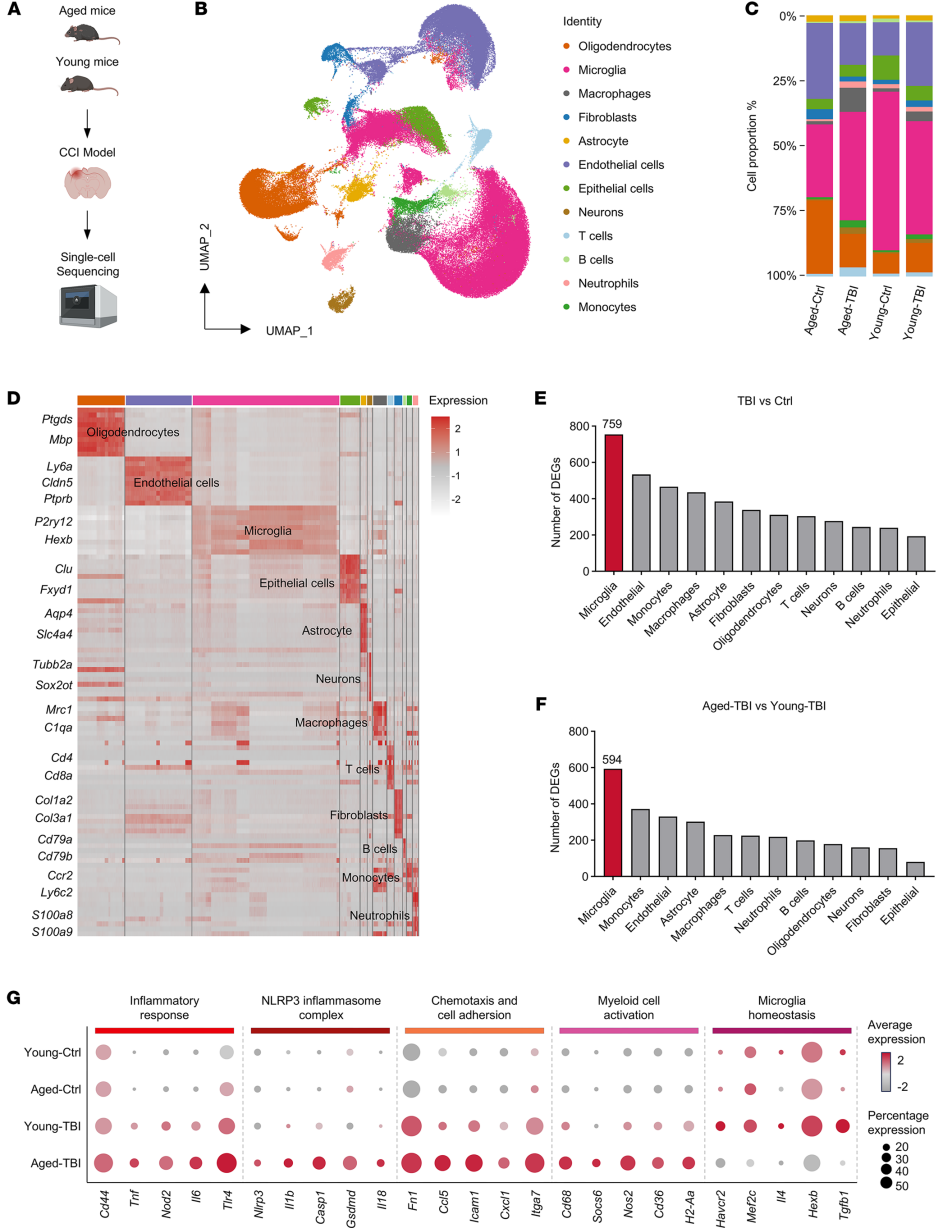
Aging enhances microglia responses after TBI. (**A**) The injured cerebral hemispheres of aged TBI mice (18 months old) and young TBI mice (4 months old) were collected, dissociated, and subjected to single-cell sequencing at day 3 after TBI. *n* = 3/group. (**B**) UMAP plot shows different cell types in the brain, distinguished by different colors. (**C**) Proportional stack plots show the percentage of different cell types in the brains from young sham-operated control group (Young-Ctrl), aged sham-operated control group (Aged-Ctrl), young TBI group (Young-TBI), and aged TBI group (Aged-TBI), distinguished by different colors. (**D**) Top 10 gene markers for each cell type; characteristic markers are labeled. (**E**) Number of DEGs between TBI groups and control groups (adjusted *P* < 0.05). Microglia showed the highest number of DEGs (759 genes). (**F**) Number of DEGs between Aged-TBI and Young-TBI groups (adjusted *P* < 0.05). Microglia showed the highest number of DEGs (594 genes). (**G**) Expression levels of selected genes in microglia and genes selected from the enriched GO terms in the set of DEGs under different conditions. The size of the dots corresponds to the proportion of cells in each condition, and the color indicates the average expression level. The schematic was generated using BioRender.

Aged patients with TBI often show poorer recovery and higher mortality than younger individuals, which may be mediated by exaggerated neuroinflammation in aged patients. To comprehensively characterize how aging affects brain inflammatory response after TBI, we used a droplet-based scRNA-seq technique to isolate cells from the injured hemisphere of aged and young mice at 72 hours after TBI modeling, which was designed to observe transcriptome alterations during the acute phase (Figure 1A). We performed unsupervised clustering and cell-type annotation based on classical gene markers. As shown in the UMAP plot, 12 distinct cell types were identified, including oligodendrocytes (19,721), microglia (61,707), macrophages (5,688), fibroblasts (3,239), astrocytes (2,735), epithelial cells (8,267), endothelial cells (27,796), neurons (1,824), T cells (2,661), B cells (1,183), granulocytes (2,384), and monocytes (2,139) (Figure 1, B and D, and Supplemental Table 1; supplemental material available online with this article; https://doi.org/10.1172/JCI196112DS1). Consistent with previous findings, we also identified the presence of circulating immune cells, such as macrophages, monocytes, neutrophils, and T and B cells, in the brain after TBI (19, 20).

In aged mice, significant microglia proliferation was observed following TBI, whereas this phenomenon was not prominent in young mice (Figure 1C and Supplemental Figure 1, A and B). Orthogonal validation via flow cytometry confirmed the findings from scRNA-seq. Specifically, TBI led to a significant increase in microglial abundance, with aged microglia showing a more pronounced expansion compared with their younger counterparts (Supplemental Figure 1, C and D).

TBI induced extensive differential gene regulation, with microglia populations showing the most pronounced changes (Figure 1E). Notably, aged microglia exhibited the highest number of differentially expressed genes (DEGs) compared with their younger counterparts after TBI, highlighting distinct age-specific transcriptional remodeling in microglia after injury (Figure 1F). The signature of aged microglia after TBI was associated with a proinflammatory phenotype, including genes involved in inflammatory responses (e.g., *Tnf*, *Nod2*, *Il6*, and *Tlr4*), NLRP3 inflammasome complex–related genes (e.g., *Nlrp3*, *Il1b*, and *Casp1*), genes involved in cellular chemotaxis and adhesion (*Fn1*, *Ccl5*, *Icam1*, and *Itga7*), and myeloid cell activation–related genes (*Cd68*, *Nos2*, and *H2-Aa*). However, genes related to microglia homeostasis (*Havcr2*, *Mef2c*, *Il4*, and *Tgf1*) were downregulated in microglia from aged TBI mice (Figure 1G).

To translate these preclinical findings to a clinical context, we obtained injured brain tissue samples from 13 young and 22 aged patients with TBI who underwent surgery and examined the proportion of microglia. Consistent with the insights obtained from murine models, aged TBI patients exhibited a more robust microglial response compared with young patients (Supplemental Figure 1, E and F). RT-qPCR of patients’ microglia revealed the distinct age-specific cytokine expression profiles. Proinflammatory cytokines *IL1B*, *IL6*, and *TNF* were significantly upregulated in microglia from aged patients, whereas the transcripts of antiinflammatory cytokines *IL4*, *IL10*, and *TGFB1* were upregulated in microglia from young patients (Supplemental Figure 1G).

### NLPR3^+^ microglia orchestrate detrimental outcomes in aged TBI.

**Figure 2:**
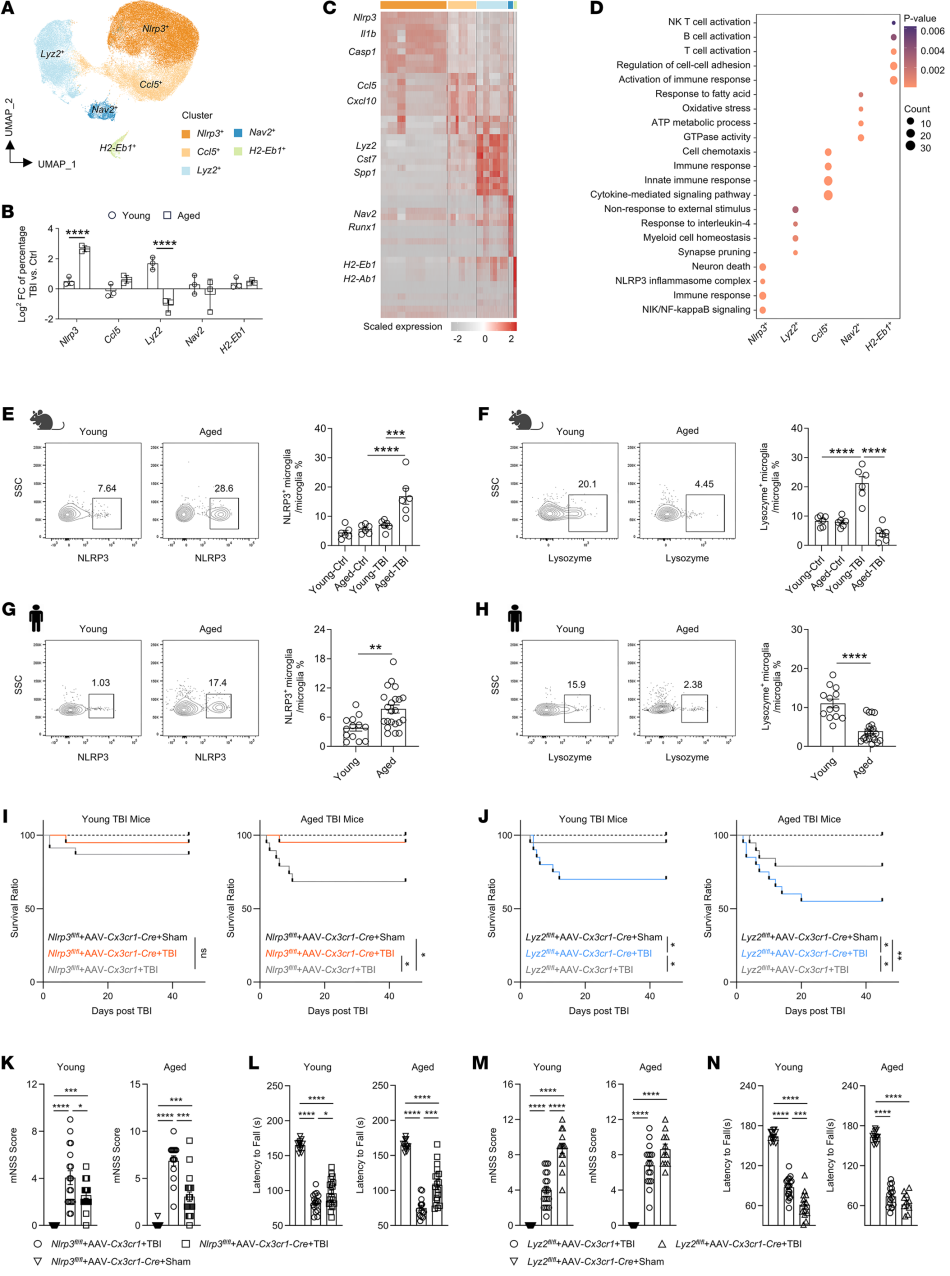
NLRP3^+^ microglia worsen the prognosis of TBI in aged mice. (**A**) UMAP plot shows the different microglia subpopulations, distinguished by different colors. *n* = 3/group. (**B**) Bar plots show the changes in the proportion of each microglial subset in young and aged mice before and after TBI. *n* = 3/group. (**C**) Gene expression heatmap (top 50 expressed) in microglia subpopulations. (**D**) Functional annotation of different microglia subpopulations. (**E** and **F**) Representative flow cytometry results and bar plots show the proportions of NLRP3^+^ and Lysozyme^+^ microglia in aged and young mice at 3 days before and after TBI. *n* = 6/group. (**G** and **H**) Representative flow cytometry results show the proportions of NLRP3^+^ and Lysozyme^+^ microglia in aged and young patients with TBI. Young, *n* = 13; aged, *n* = 22. (**I**) Survival curves of aged and young mice after AAV-*Cx3cr1*-*Cre* KO of *Nlrp3* in microglia after TBI (start: *n*_AAV-*Cx3cr1*-*Cre*-Sham_ = 12, *n*_AAV-*Cx3cr1*-*Cre*-TBI_ = 20, and *n*_AAV-*Cx3cr1*-TBI_ = 20). (**J**) Survival curves of aged and young mice after AAV-*Cx3cr1*-*Cre* KO of *Lyz2* in microglia after TBI (start: *n*_AAV-*Cx3cr1*-*Cre*-Sham_ = 12, *n*_AAV-*Cx3cr1*-*Cre*-TBI_ = 20, and *n*_AAV-*Cx3cr1*-TBI_ = 20). (**K**) mNSSs after 45 days of TBI under different conditions related to **I**. Young: *n*_AAV-*Cx3cr1*-*Cre*-Sham_ = 12, *n*_AAV-*Cx3cr1*-*Cre*-TBI_ = 19, and *n*_AAV-*Cx3cr1*-TBI_ = 18. Aged: *n*_AAV-*Cx3cr1*-*Cre*-Sham_ = 12, *n*_AAV-*Cx3cr1*-*Cre*-TBI_ = 19, and *n*_AAV-*Cx3cr1*-TBI_ = 14. (**L**) Rotarod test after 45 days of TBI under different conditions related to **I**. The number of animals in each group is consistent with **K**. (**M**) mNSSs after 45 days of TBI under different conditions related to **J**. Young: *n*_AAV-*Cx3cr1*-*Cre*-Sham_ = 12, *n*_AAV-*Cx3cr1*-*Cre*-TBI_ = 14, *n*_AAV-*Cx3cr1*-TBI_ = 19. Aged: *n*_AAV-*Cx3cr1*-*Cre*-Sham_ = 12, *n*_AAV-*Cx3cr1*-*Cre*-TBI_ = 11, *n*_AAV-*Cx3cr1*-TBI_ = 16. (**N**) Rotarod test after 45 days of TBI under different conditions related to **J**. The number of animals in each group is consistent with **M**. Data are presented as mean ± SEM. *P < 0.05, **P < 0.01, ***P < 0.001, ****P < 0.001. Statistical analyses were performed using unpaired 2-tailed Student’s *t* test (**G** and **H**), 2-way ANOVA followed by Tukey’s post hoc test (**E**, **F**, and **K**–**N**), and Kaplan-Meier survival analysis (**I** and **J**). The schematic was generated using BioRender.

To decipher how aging affects the transcriptional profile of microglia after TBI, we clustered microglia from different samples and observed substantial heterogeneity among microglia after TBI. The UMAP plot shows 5 states consisting of *Nlrp3*^+^ (*Nlrp3*^+^*Il1b*^+^*Casp1*^+^), *Lyz2*^+^ (*Lyz2*^+^*Cst7*^+^*Spp1*^+^), *Ccl5*^+^ (*Ccl5*^+^*Cxcl10*^+^), *Nav2*^+^ (*Nav2*^+^*Runx1*^+^), and *H2-Eb1*^+^ (*H2-Eb1*^+^*H2-Ab1*^+^) microglia after TBI (Figure 2, A–C). For instance, *H2-Eb1*^+^ microglia exhibit high expression of MHC-II molecules, representing a population of microglia that may have antigen-presenting capacity. We identified clusters that may represent features of the inflammatory response (*Nlrp3*^+^) and specific chemotaxis (*Ccl5*^+^). Some subsets of our data show partial concordance with previously published reports; characteristic genes of *Lyz2*^+^ microglia, such as *Cst7* and *Spp1*, are widely identified in stage 2 DAMs (15, 21), and the sensitivity of *Lyz2*^+^ microglia to IL-4 suggests its important role in limiting neuroinflammation (Figure 2D). In summary, our microglial atlas not only captures known microglia subpopulation features, but also reveals potentially uncharacterized microglia subpopulations.

The *Nlrp3*^+^ cluster representing neuroinflammatory responses was upregulated in aged microglia after TBI, while the *Lyz2*^+^ cluster representing tissue repair and myeloid homeostasis showed enhanced enrichment in young microglia (Figure 2B). To determine the representativeness of the markers for each microglia cluster suggested by scRNA-seq, we used the cluster 0–specific marker NLRP3 and the cluster 1–specific marker Lysozyme (the protein encoded by the *Lyz2* gene) to label different clusters of microglia. The proportion of NLRP3^+^ microglia infiltrating the injured area was higher in aged TBI mice, whereas the opposite was true for Lysozyme^+^ microglia (Figure 2, E and F, and Supplemental Figure 2). To extend this finding to a broader spectrum of brain injury states, we validated it in mice models of repetitive mild TBI (rmTBI). In rmTBI mice, our findings still hold, with aged mice characterized by enhanced NLRP3^+^ microglia responses, whereas young microglia exhibited increased Lysozyme^+^ phenotype after brain injury (Supplemental Figure 3, A–D). Flow cytometry and representative immunofluorescence results of different ages of patients with TBI corroborated the insights generated from TBI mice that aged microglia responses after TBI were dominated by the NLRP3^+^ cluster, whereas young microglia responses were dominated by the Lysozyme^+^ cluster (Figure 2, G and H, Supplemental Figure 4, and Supplemental Figure 5).

To investigate the effect of different microglia subpopulations on the prognosis of patients with TBI, we used the AAV-*Cx3cr1*-*Cre* virus to knock out microglial *Nlrp3* in *Nlrp3*^fl/fl^ mice after TBI. Through Ai9 reporter mice, we confirmed the specificity of this microglia-targeting strategy (Supplemental Figure 6, A–E). Flow cytometry confirmed the specific deletion of NLRP3 in microglia (Supplemental Figure 7, A and B). After *Nlrp3* KO in microglia, TBI mice exhibited lower mortality and better neurological recovery (lower modified Neurological Severity Score [mNSS] and prolonged latency to fall from the rotarod test) (Figure 2, I, K, and L). Furthermore, we explored the efficacy of MCC950, a selective inhibitor of NLRP3. MCC950 administration reduced mortality and NLRP3^+^ microglia responses in the acute-phase aged TBI mice (Supplemental Figure 8, A–D). Suppression of neuroinflammation after MCC950 treatment significantly improved neurological recovery in aged TBI mice (Supplemental Figure 8E). Interestingly, we also found that MCC950 prevented the brain infiltration of circulating lymphocytes after TBI by suppressing the transcription of lymphocyte chemotactic factor in microglia (Supplemental Figure 8, F–I).

In contrast, after *Lyz2* KO in microglial, both aged and young TBI mice exhibited higher mortality and worse neurologic prognosis (higher mNSSs and shorter latency to fall from the rotarod tests) (Figure 2, J, M, and N, and Supplemental Figure 7C). Together, these findings suggest that the age-specific signature of enhanced NLRP3^+^ microglial responses coupled with impaired Lysozyme^+^ microglial function may contribute to the poorer prognosis of aged mice following TBI.

### Metabolic reprogramming underlies proinflammatory phenotype of NLRP3^+^ microglia.

**Figure 3:**
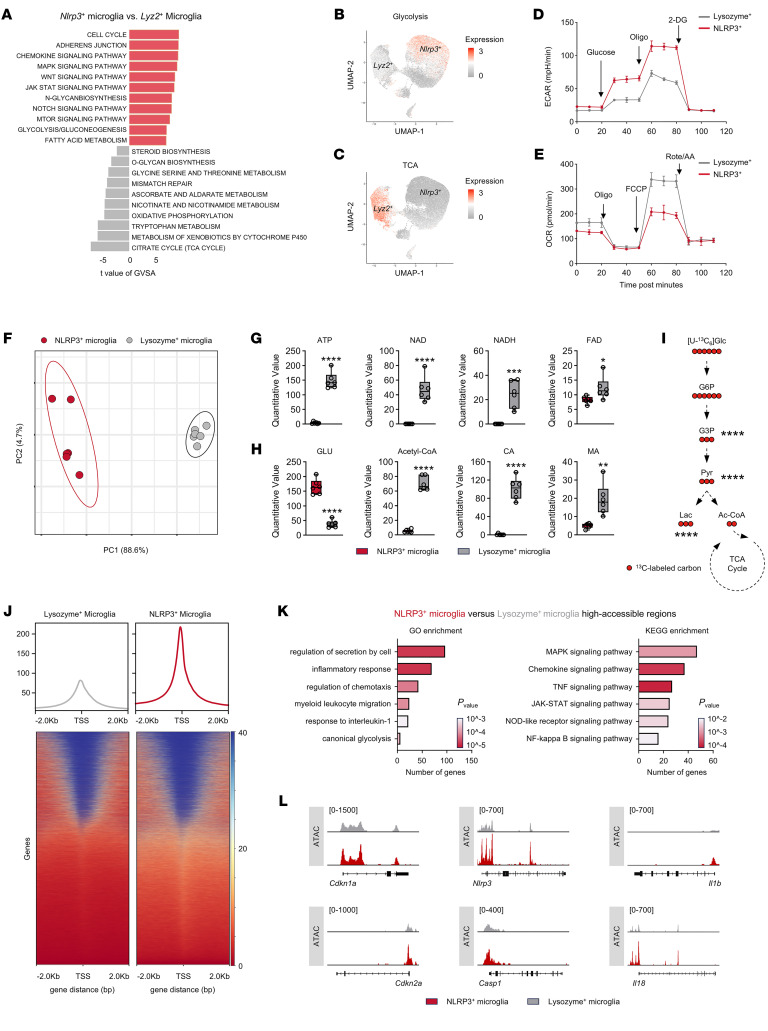
Enhanced aging-induced glycolysis induces the generation of NLRP3^+^ microglia. (**A**) GVSA enrichment of differential biological functions between Nlrp3^+^ and Lyz2^+^ microglia. (**B** and **C**) Gene score feature plots in microglia using representative genes involved in glycolysis and the TCA cycle. (**D**) Line plot shows the ECAR of NLRP3^+^ and Lysozyme^+^ microglia sorted from TBI mice. *n* = 3/group. (**E**) Line plot shows the OCR of NLRP3^+^ and Lysozyme^+^ microglia sorted from TBI mice. *n* = 3/group. (**F**) Distribution of untargeted metabolomics of NLRP3^+^ and Lysozyme^+^ microglia in principal component analysis PCA space. *n* = 6/group. (**G** and **H**) Bar plots show the relative abundance of metabolites related to energy metabolism and the TCA cycle in NLRP3^+^ and Lysozyme^+^ microglia. *n* = 6/group. (**I**) NLRP3^+^ microglia from aged TBI mice show significantly higher ^13^C-labeling efficiency in glyceraldehyde-3-phosphate (G3P), pyruvate, and lactate compared with Lysozyme^+^ microglia from young TBI mice. *n* = 4/group. (**J**) Density heatmap of differential open chromatin regions in NLRP3^+^ and Lysozyme^+^ microglia. *n* = 3/group. (**K**) GO (left) and KEGG (right) enrichment of genes associated with higher chromatin accessibility in NLRP3^+^ microglia compared with Lysozyme^+^ microglia. (**L**) Integrative Genomics Viewer plots show chromatin accessibility of *Cdkn1a* (*p16*), *Cdkm2a* (*p21*), *Nlrp3*, *Casp1*, *Il1b*, and *Il18* in NLRP3^+^ and Lysozyme^+^ microglia. Data are presented as mean ± SEM. *P < 0.05, **P < 0.01, ***P < 0.001, ****P < 0.001. Statistical analyses were performed using unpaired 2-tailed Student’s *t* test (**G** and **H**).

To unravel the mechanisms underlying the enhanced proinflammatory response of aged microglia, we performed gene set variation analysis (GSVA) comparing NLRP3^+^ and Lysozyme^+^ microglia. GSVA indicated that NLRP3^+^ microglia upregulate inflammation-related signaling pathways (Figure 3A). Notably, NLRP3^+^ microglia may increase their dependence on glycolysis, while the energy metabolism of young microglia may be dependent on the TCA cycle and OXPHOS. This observation was validated by analyzing the expression of genes associated with these target metabolic pathways by AddModuleScore (Figure 3, B and C). Subsequently, we detected real-time changes in extracellular acidification rate (ECAR) and oxygen consumption rate (OCR) in NLRP3^+^ microglia and Lysozyme^+^ microglia by Seahorse. We observed aging-induced increases in ECAR and decreases in OCR, representing the enhancement of cellular glycolysis along with the weakening of TCA, which was consistent with our scRNA-seq analysis (Figure 3, D and E).

Microglial function is closely related to the metabolic state (16). To further characterize age-related metabolic differences in microglia after TBI, we purified microglia from aged and young TBI mice via FACS and performed untargeted metabolomics analysis. Aging drove distinct metabolic remodeling in microglia, with NLRP3^+^ microglia separating from Lysozyme^+^ microglia along principal component 1, which accounted for 88.6% of the variance (Figure 3F). We observed that NADH and FADH_2_ equivalents for ATP generation by the electron transport chain were significantly decreased in NLRP3^+^ microglia (Figure 3G). For this reason, we explored the metabolites associated with TCA activity in microglia. Notably, we detected a decrease in most TCA intermediates in NLRP3^+^ microglia, including acetyl-CoA, α-ketoglutarate, citrate, and malate (Figure 3H).

To directly validate the glycolytic flux in microglial subsets, we performed U-^13^C_6_-glucose metabolic tracing experiments and analyzed the isotope labeling patterns of glycolytic intermediates. After incubating sorted microglia with U-^13^C_6_-glucose for 60 minutes, NLRP3^+^ microglia from aged TBI mice showed significantly higher ¹³C-labeling efficiency in glyceraldehyde-3-phosphate, pyruvate, and lactate compared with Lysozyme^+^ microglia from young TBI mice (Figure 3I). Collectively, these data suggested that metabolic reprogramming in aging-associated NLRP3^+^ microglia strongly favors glycolytic dependence.

Previous studies have suggested that metabolic reprogramming of microglia may be associated with altered chromatin accessibility (17, 22). We hypothesized that the glycolytic preference of NLRP3^+^ microglia drives the acquisition of proinflammatory chromatin features. To test this, we performed ATAC-seq on NLRP3^+^ microglia versus Lysozyme^+^ microglia to reveal the link between metabolic reprogramming and the inflammatory chromatin landscape and observed increased chromatin accessibility in NLRP3^+^ microglia (Figure 3J). Functional enrichment analysis revealed that regions of increased accessibility in the chromatin of NLRP3^+^ microglia were associated with upregulation of inflammatory responses and inflammation-related signaling pathways (Figure 3K). Importantly, compared with Lysozyme^+^ microglia, NLRP3^+^ microglia acquired specific peaks in genes associated with cellular senescence *(Cdkn1a* and *Cdkn2a*) and the NLRP3 inflammasome complex (*Nlrp3*, *Casp1*, *Il1b*, and *Il18*) (Figure 3L). RT-qPCR further confirmed that NLRP3^+^ microglia enhanced transcription of senescence-associated secretory phenotype markers, including *Cdkn1a*, *Cdkn2a*, *Il1b*, and *Il18* (Supplemental Figure 9A). Overall, our data connect energy metabolism rewiring with altered chromatin accessibility, explaining how aging upregulates the proportion of NLRP3^+^ microglia after TBI.

### ELF1 governs the transcriptional features of age-associated NLRP3^+^ microglia formation.

**Figure 4:**
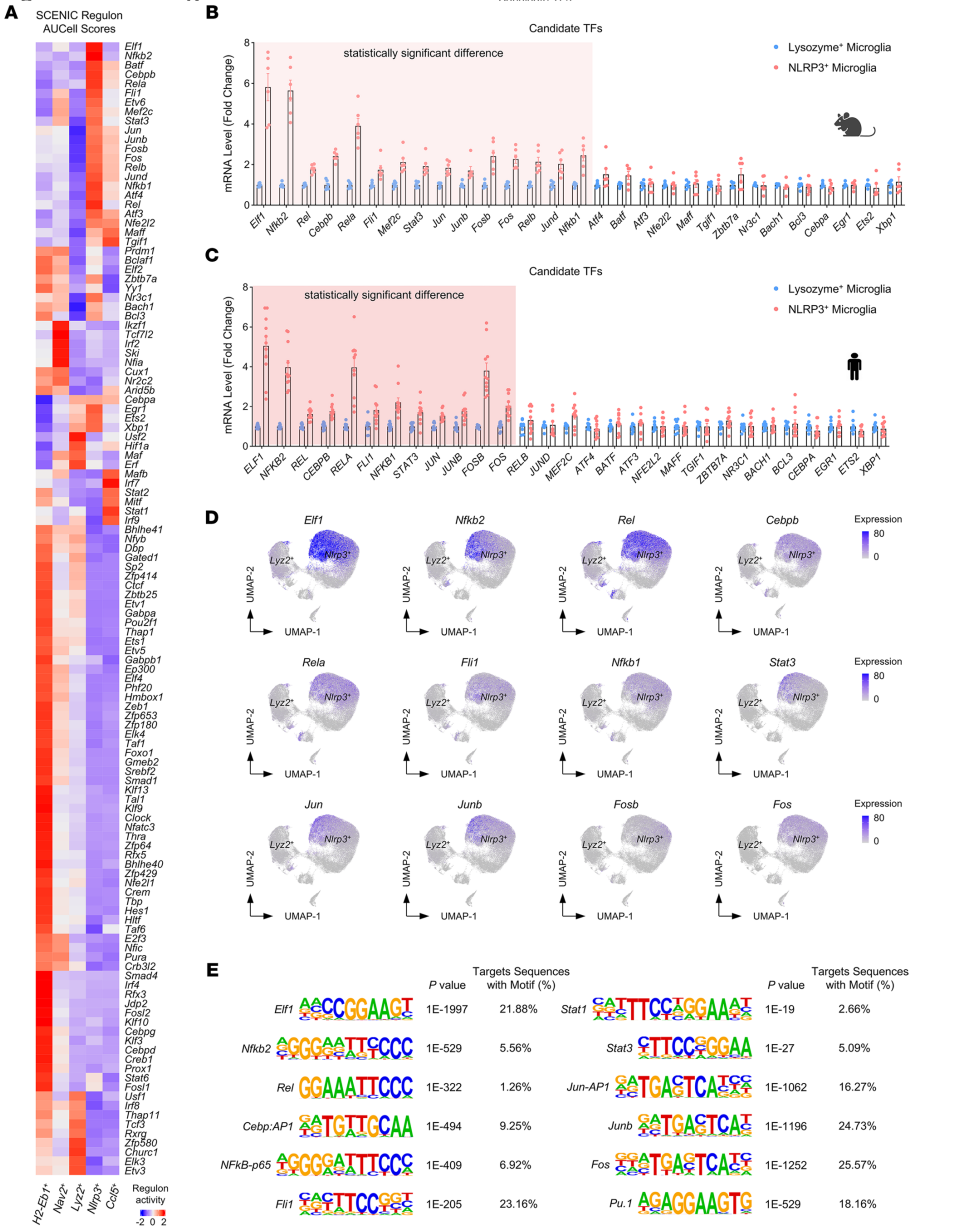
Inflammation-related TFs are associated with the formation of NLRP3^+^ microglia. (**A**) SCENIC heatmap of TF-regulated microglia subpopulations based on scRNA-seq. (**B**) Bar plots show the qPCR results of 29 alternative TFs in NLRP3^+^ and Lysozyme^+^ microglia sorted from TBI mice. *n* = 6/group. (**C**) qPCR analysis of 29 alternative TFs in NLRP3^+^ and Lysozyme^+^ microglia sorted from patients with TBI. *n*_NLRP3+_ = 11, *n*_Lysozyme+_ = 9. (**D**) Feature plots show the expression of 12 differential TFs coregulated in human and mouse NLRP3^+^ microglia in different subpopulations of microglia. (**E**) Tables illustrating the ATAC-seq motif enrichment analysis for NLRP3^+^ microglia. Data are presented as mean ± SEM. Statistical analyses were performed using unpaired 2-tailed Student’s *t* test (**B** and **C**). The schematic was generated using BioRender.

We found that aging-induced impairment of energy metabolism may regulate the inflammatory state of microglia across different age groups by modulating chromatin accessibility. We next sought to identify the intracellular regulators that drive the generation of NLRP3^+^ microglia and employed the SCENIC algorithm to dissect the key molecular mechanisms. We observed that a wide range of inflammatory response–associated transcriptional regulators (TFs) were involved in the development of NLRP3^+^ microglia. Specifically, genes related to the NF-κB signaling pathway (*Rela* and *Relb*), the MAPK signaling pathway (*Fos* and *Jun*), and the JAK/STAT signaling pathway (*Stat1* and *Stat3*) were found to be implicated in the formation of NLRP3^+^ microglia (Figure 4A).

To further characterize NLRP3^+^ microglia, we validated 29 human homologs of SCENIC-identified differentially expressed TFs in mouse and human NLRP3^+^ microglia. Twelve TFs (*ELF1*, *NFKB2*, *REL*, *CEBPB*, *RELA*, *FLI1*, *NFKB1*, *STAT3*, *JUN*, *JUNB*, *FOSB*, and *FOS*) were concordantly upregulated in both species, emerging as potential regulators of NLRP3^+^ microglial formation (Figure 4, B and C). Feature plots confirmed these TFs were barely expressed in Lysozyme^+^ microglia (Figure 4D). Next, we performed motif enrichment of these selected TFs using ATAC-seq. The results showed that these TFs, which are known to play a key role in mediating the inflammatory cascade response, were enriched in NLRP3^+^ microglia (Figure 4E).

**Figure 5:**
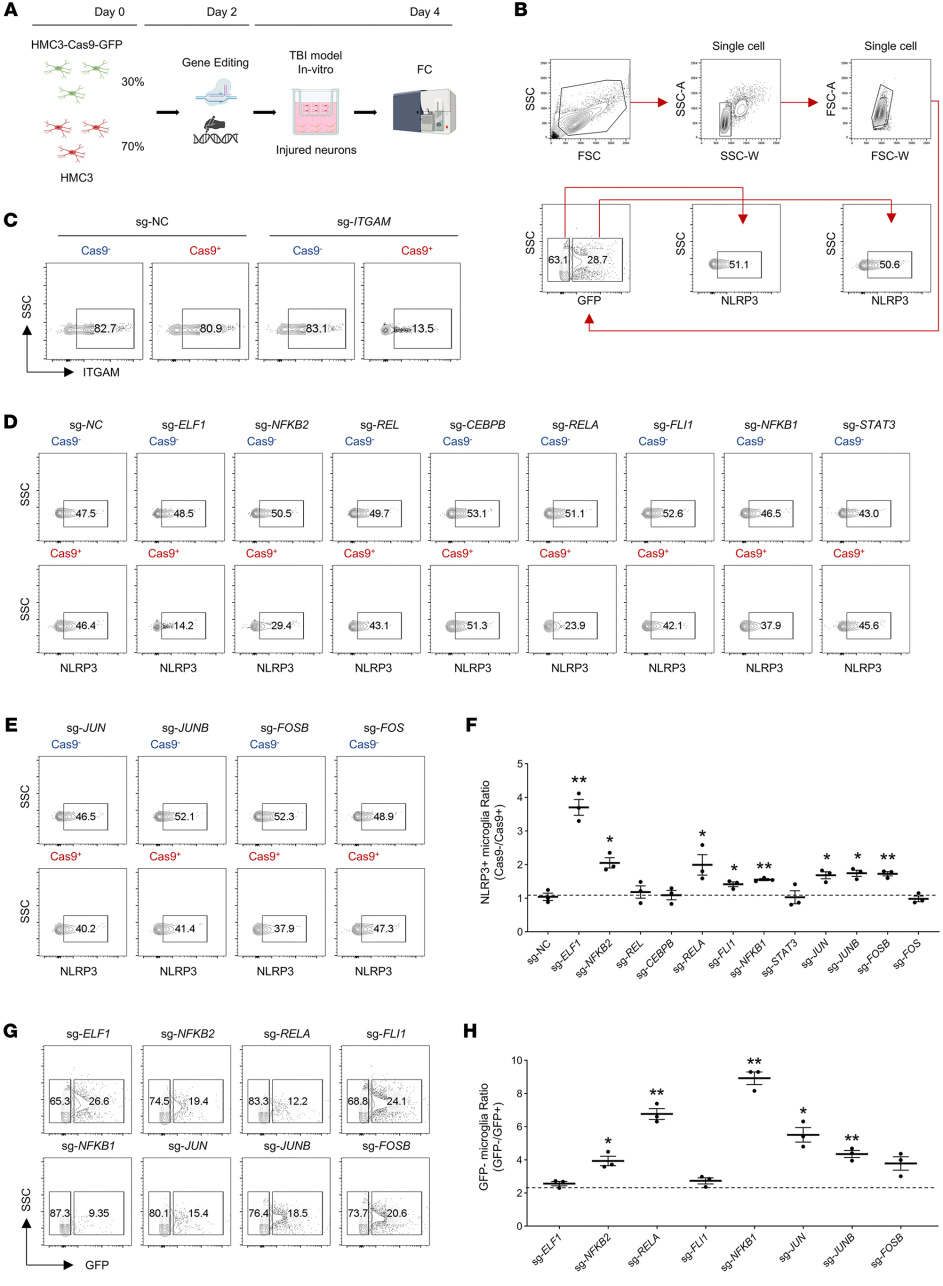
ELF1 predominantly regulates the formation of NLRP3^+^ microglia. (**A**) Microglia expressing Cas9-GFP were admixed with normal microglia at a certain ratio, and flow cytometry (FC) was performed after 2 days of cocultivation with broken neurons using sgRNA-induced ablation of the target gene. (**B**) Flow cytometry strategy for detecting NLPR3^+^ microglia in GFP^–^ (Cas9^–^) and GFP^+^ (Cas9^+^) microglia. (**C**) Gene editing efficiency of sgRNAs was assayed using sg-*ITGAM*. (**D**–**F**) Changes in the proportion of NLRP3^+^ microglia after ablation of 12 alternative TFs. *n* = 3/group. (**G** and **H**) Changes in the proportion of GFP^–^/GFP^+^ microglia indicating the cell survival ratio of GFP^+^ microglia after ablation of 8 alternative TFs. *n* = 3/group. Data are presented as mean ± SEM. *P < 0.05, **P < 0.01. Statistical analyses were performed using unpaired 2-tailed Student’s *t* test (**F** and **H**). The schematic was generated using BioRender.

To identify the TFs that mainly drive human NLRP3^+^ microglia formation, we infected a preconstructed Cas9-GFP human microglia cell line (HMC3) with sgRNAs targeting each TF. These cells were mixed with normal HMC3 cells and cocultured with needle scratch–injured SH-SY5Y neurons (Figure 5A). We quantified the live GFP^+^/GFP^–^ microglia ratio and verified genome editing efficiency using *ITGAM*-specific sequences (Figure 5, B and C). We identified 8 TFs (*ELF1*, *NFKB2*, *RELA*, *FLI1*, *NFKB1*, *JUN*, *JUNB*, and *FOSB*) that significantly promote NLRP3^+^ microglia formation; *ELF1* ablation exhibited the strongest inhibitory effects on NLRP3^+^ microglia generation and IL-1β secretion (Figure 5, D–F, and Supplemental Figure 11, A and B). Notably, ablation of *ELF1* in microglia had little effect on the microglial viability (Figure 5, G and H). In Cas9^+^ HMC3 monocultures, 3 distinct *ELF1*-targeting sgRNAs reduced NLRP3^+^ microglia formation, ruling out off-target effects (Supplemental Figure 10, A and B).

To clarify the specific role of ELF1 in NLRP3^+^ microglia formation, we established a THP-1-Cas9-GFP cell line and *ELF1* KO. While *ELF1* ablation attenuated TBI-induced IL-1β secretion in THP-1 cells, the effect was weaker than in the HMC3 cell line. Quantitative comparison further confirmed that ELF1 plays a far more prominent role in regulating inflammation in microglia than peripheral myeloid cells (Supplemental Figure 11, C–H).

### Ablation of Elf1 reverses aging-related microglia dysfunction and improves the outcome of TBI mice.

To further investigate ELF1’s in vivo regulation of microglial inflammatory responses, we crossed *Elf1*^fl/fl^ mice with *Cx3cr1*^creERT2^ mice (Cre-only) and constructed *Elf1*^fl/fl^ *Cx3xr1*^CreERT2^ (cKO) mice to selectively ablate *Elf1* expression in CX3CR1^+^ cells. To rule out a potential effect of *Elf1* deletion on brain development, *Elf1* was deleted only after tamoxifen administration. Specifically, tamoxifen induced ablation of *Elf1* in cells expressing *Cx3cr1*, including microglia and peripheral myeloid cells. Notably, bone marrow–derived peripheral myeloid cells were continuously replenished by newborn CX3CR1^+^ cells, leading to almost complete renewal of the initial *Elf1*-KO macrophage population within 30 days. In contrast, microglia underwent self-renewal and retained lifelong *Elf1* ablation (Supplemental Figure 12A).

To verify the efficiency of removing microglial *Elf1*, on day 35 after tamoxifen administration, we used FACS to isolate microglia (CD45^int^CD11b^+^TMEM119^+^), other brain cells (CD45^–^CD11b^–^), and circulation myeloid cells (CD45^+^CD11b^+^). As expected, quantitative analysis of *Elf1* mRNA in these FACS-isolated microglia showed that *Elf1* was virtually eliminated in microglia, whereas *Elf1* expression was unaffected in macrophages and other brain cells (Supplemental Figure 12, B and C).

**Figure 6:**
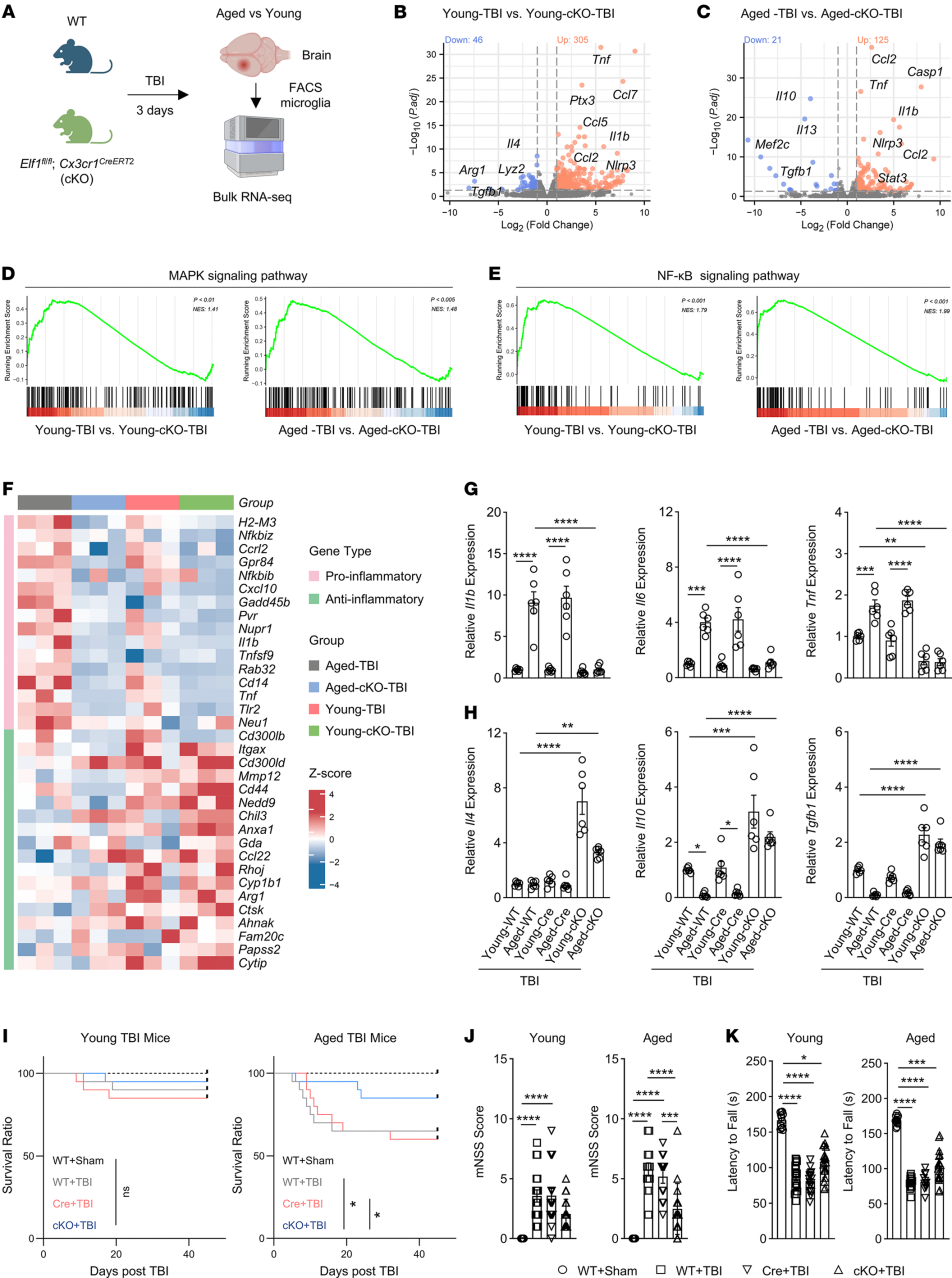
In vivo ablation of ELF1 improves the survival and neurological recovery of mice after TBI. (**A**) Brain tissues from WT and cKO mice 3 days after TBI were collected, and transcriptome sequencing was performed after FACS enrichment of microglia. (**B**) Volcano plot of microglia DEGs between Young-TBI mice and Young-cKO-TBI mice. *n* = 3/group. (**C**) Volcano plot of microglia DEGs between Young-TBI mice and Young-cKO-TBI mice. *n* = 3/group. (**D**) GSEA of DEGs in the MAPK signaling pathway between Young-TBI mice and Young-cKO-TBI mice. *n* = 3/group. (**E**) GSEA of DEGs in the NF-κB signaling pathway between Young-TBI and Young-cKO-TBI mice. *n* = 3/group. (**F**) Proinflammatory and antiinflammatory expression profiles of different groups of microglia. Each column represents 1 sample. Data are expressed as *z* scores representing the mean and SD normalized expression levels for each gene (row). (**G** and **H**) qPCR detection of pro- and antiinflammatory cytokines in different groups of microglia. *n* = 6/group. (**I**) Survival curves of 4 different mice conditions within 45 days of TBI (left, young group; right, aged group; start: *n*_WT+Sham_ = 12, *n*_WT+Sham_ = 20, *n*_Cre+TBI_ = 20, and *n*_cKO+TBI_ = 20). (**J** and **K**) mNSS and rotarod test of 4 different mice conditions after 45 days of TBI (young group, *n*_WT+Sham_ = 12, *n*_WT+Sham_ =18, *n*_Cre+TBI_ = 17, and *n*_cKO+TBI_ = 19; aged group, *n*_WT+Sham_ = 12, *n*_WT+Sham_ = 13, *n*_Cre+TBI_ = 12, and *n*_cKO+TBI_ = 17). Data are presented as mean ± SEM. *P < 0.05, **P < 0.01, ***P < 0.001, ****P < 0.0001. Statistical analyses were performed using 2-way ANOVA followed by Tukey’s post hoc test (**G**, **H**, **J**, and **K**) and Kaplan-Meier survival analysis (**I**). The schematic was generated using BioRender.

To confirm the protective functions of *Elf1*-cKO after TBI, we performed bulk RNA-seq and compared the differences between purified microglia 3 days after TBI (Figure 6A). Ablation of *Elf1* induced upregulation of antiinflammation-related genes such as *Arg1*, *Tgfb1*, *Lyz2*, and *Sall1* in microglia, while inflammation-related genes such as *Tnf*, *Ccl2*, *Nlrp3*, *Il1b*, and *Casp1* were downregulated (Figure 6, B and C). In addition, ablation of *Elf1* mediated the downregulation of the inflammatory, MAPK, and NF-κB signaling pathways in aged and young microglia (Figure 6, D and E) and downregulated the proportion of NLRP3^+^ microglia and upregulated the proportion of Lysozyme^+^ microglia after TBI (Supplemental Figure 12, D–G).

To further characterize the inflammatory signature of *Elf1*-deficient microglia, we compared our TBI-induced DEG dataset with pro- and antiinflammatory markers from the NCBI Gene Expression Omnibus public database (GSE69607). This analysis revealed that *Elf1*-ablated microglia exhibited a distinct inflammatory profile after TBI, with reduced proinflammatory gene expression and elevated antiinflammatory gene expression (Figure 6F). RT-qPCR confirmed the proinflammatory markers (*Il1b*, *Il6*, and *Tnf*) were downregulated and antiinflammatory markers (*Il4*, *Il10*, and *Tgfb1*) were upregulated in cKO-TBI compared with WT and Cre controls (Figure 6, G and H). Modulation of the inflammatory response brought about significant improvements in survival and behavioral performance, as evidenced by a significant decrease in mortality in aged TBI mice, a decrease in mNSS, and a prolongation of the latency to fall from the rotarod test (Figure 6, I–K).

### Targeting ELF1 with Imeglimin confers neuroprotection in TBI.

**Figure 7:**
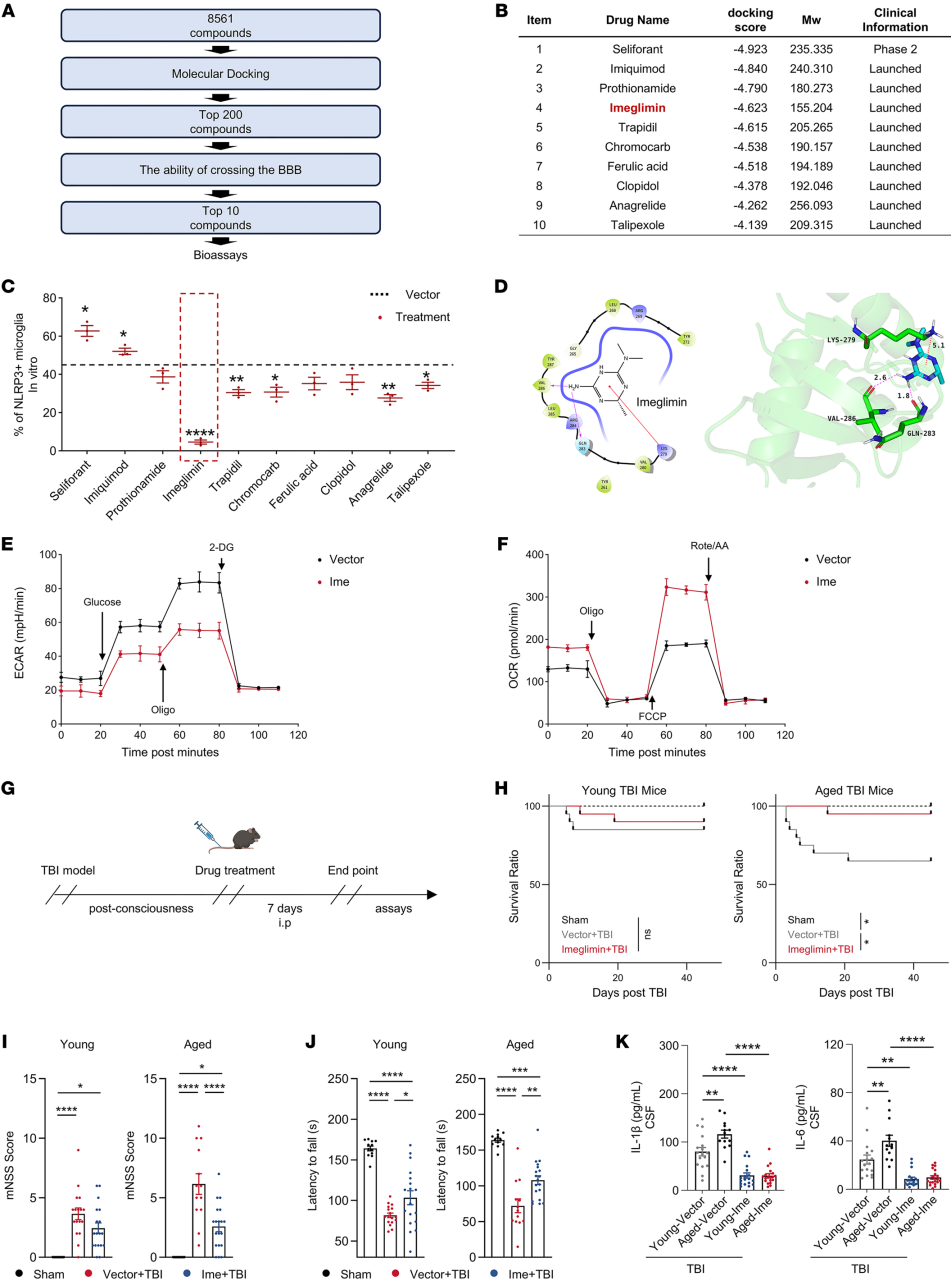
Imeglimin treatment promotes recovery in mice with TBI. (**A**) High-throughput virtual screening was performed on 8,561 potential ELF1 inhibitor compounds approved by the FDA or already in clinical use. 200 compounds with the highest docking scores were screened after sequential SP and XP docking analysis. Finally, 10 compounds were screened out of all 200 compounds with the highest potential to cross the blood-brain barrier (BBB). (**B**) Docking scores, molecular weights, and clinical information for the 10 compounds selected. (**C**) Ratio of NLRP3^+^ microglia after Imeglimin treatment (HMC3). *n* = 3/group. (**D**) Chemical structure of Imeglimin and in silico docking to the active pocket of the human ELF1 protein. (**E** and **F**) ECAR and OCR of Imeglimin-treated microglia cell line (HMC3) and vector controls. *n* = 3/group. (**G**) Flow chart of Imeglimin in vivo treatment of mice with TBI. (**H**) Survival curves of Imeglimin-treated mice versus vector control mice after 45 days of TBI (left, young group; right, aged group; start: *n*_Sham_ = 12, *n*_Vector+Sham_ = 20, and *n*_Ime+TBI_ = 20). (**I** and **J**) mNSS (**I**) and rotarod test (**J**) between Imeglimin-treated and vector control mice after 45 days of TBI (young group, *n*_Sham_ = 12, *n*_Vector+TBI_ = 17, and *n*_Ime+TBI_ = 18; aged group, *n*_Sham_ = 12, *n*_Vector+TBI_ = 13, and *n*_Ime+TBI_ = 19). (**K**) Concentrations of IL-1β and IL-6 in the cerebrospinal fluid of Imeglimin-treated and vector control mice after 45 days of TBI (*n*_Young-vector_ = 17, *n*_Aged-vector_ = 13, *n*_Young-Ime_ = 18, and *n*_Aged+Ime_ = 19). Data are presented as mean ± SEM. *P < 0.05, **P < 0.01, ****P < 0.0001. Statistical analyses were performed using unpaired 2-tailed Student’s *t* test (**C**), 2-way ANOVA followed by Tukey’s post hoc test (**I**–**K**), and Kaplan-Meier survival analysis (**H**). The schematic was generated using BioRender.

Currently, there are no commercially available ELF1 inhibitors. Therefore, we performed a structure-based high-throughput virtual screening of 8,561 compounds approved by the FDA or already in clinical use for potential ELF1 inhibitors. After successive standard and high-precision docking analyses, the 200 compounds with the highest docking scores were selected. After comprehensively analyzing the molecular weight, lipid solubility, and affinity to protein components in the blood-brain barrier of the 200 compounds, we screened the 10 compounds most likely to cross the blood-brain barrier for subsequent experiments (Figure 7, A and B).

Among all 10 alternative compounds, Imeglimin, a newly marketed antidiabetic agent with a good safety profile (23), showed the strongest inhibitory effect on NLRP3^+^ microglia formation (Figure 7C and Supplemental Figure 13, A–D). Molecular docking showed that Imeglimin binds to the ATP pocket of the ELF1 protein through 3 hydrogen bonds (formed with Val286, Gln283, and Lys279; Figure 7D). Imeglimin modulated mitochondrial dysfunction of microglia and improved the aerobic respiration of microglia, as indicated by decreased ECAR and increased OCR (Figure 7, E and F). Based on a study of Imeglimin in ischemic brain injury, we determined the in vivo therapeutic concentration of Imeglimin to be 135 μg/kg/day (24). After in vivo treatment at a concentration of 135 μg/kg for 7 days, Imeglimin increased the survival rate of mice with TBI within 45 days (Figure 7, G and H) and decreased the proportion of NLRP3^+^ microglia in the brain after injury (upregulated the proportion of Lysosome^+^ microglia) (Supplemental Figure 14, A–D). Imeglimin improved the neurological recovery and decreased the concentrations of proinflammatory cytokines IL-6 and IL-1β in the cerebrospinal fluid of mice after TBI (Figure 7, I–K). Interestingly, Imeglimin treatment reduced lymphocyte burden in the brains of mice with TBI and decreased the transcription of senescence-related markers Cdkn1a and Cdkn2a in microglia (Supplemental Figure 15, A–D).

To better understand how Imeglimin functions by blocking ELF1 rather than off-target effects, we constructed 3 HMC3 cell lines (WT, *ELF1*-KO, and *ELF1*-KO with Imeglimin treatment) and performed in vitro TBI modeling (Supplemental Figure 16A). Administration of Imeglimin in *ELF1*-KO microglia did not continue to reduce the proportion of NLRP3^+^ microglia and did not improve ECAR and OCR in *ELF1*-KO microglia (Supplemental Figure 16, B, E, and F). To further analyze this, we constructed 3 HMC3 cell lines (WT, *ELF1*-overexpressing [*ELF1*-OE], and *ELF1*-OE with Imeglimin treatment) (Supplemental Figure 16C). The proportion of NLRP3^+^ microglia was significantly elevated after *ELF1* overexpression, whereas Imeglimin reversed this result and achieved a significant improvement even compared with WT control. Administration of Imeglimin in the *ELF1*-OE group significantly restored microglial aerobic respiration with improved ECAR and OCR (Supplemental Figure 16, D, G, and H). The results of the in vivo experiments corroborated the findings from the in vitro experiments. That is, after *Elf1* KO, Imeglimin could not further reduce the proportion of NLRP3^+^ microglia in the brain after TBI (Supplemental Figure 16, I and J). The above findings suggest that Imeglimin exerts its pharmacological effects mainly through blocking ELF1 rather than off-target effects.

Regarding the safety of Imeglimin treatment, we found that it did not cause a decrease in blood glucose of sham mice, and the rise in blood glucose after TBI was suppressed by Imeglimin treatment (Supplemental Figure 17A). Except for blood glucose, Imeglimin treatment did not cause blood pressure fluctuations in TBI mice (Supplemental Figure 17, B and C). Histologic sections of each major organ did not indicate obviously toxicological effects (Supplemental Figure 17D).

## Discussion

In this study, we used scRNA-seq to reveal the characteristics of the brain immune microenvironment in the acute phase of TBI in aged and young mice. Myeloid immune responses dominated the acute phase after TBI and were particularly severe in aged mice. Among all myeloid immune cells, the number and proportion of microglia showed significant differences between aged and young mice after TBI. After downscaling the aggregates of microglia from different samples, we identified the 2 clusters of microglia most associated with TBI prognosis: the *Nlrp3*^+^ cluster (worse prognosis, higher prevalence in the aged microglia) and the *Lyz2*^+^ cluster (improved prognosis, higher prevalence in the younger microglia). Although we validated the findings in the brain tissues from young and aged patients with TBI, the small sample size and the heterogeneity of injury severity, treatment, tissue sampling, sample processing, and many other factors may confound the results. In addition, peripheral immune cells respond to chemokines and infiltrate the brain after TBI. Previous studies have shown that T cells and neutrophils interact with microglia and worsen TBI prognosis (25–28). These microglia subpopulations are disease specific and defined in the context of a comprehensive set of considerations, including spatiotemporal context, metabolic profile, and biological function.

Furthermore, several markers could provide a glimpse of the potential functions of *Nlrp3*^+^ and *Lyz2*^+^ microglia. Specifically, *Nlrp3* may promote the inflammatory response after TBI, whereas *Cst7* and *Spp1*, which are highly expressed in the *Lyz2*^+^ microglia, are widely recognized in stage 2 DAMs to inhibit neurodegeneration by enhancing microglia phagocytosis (15). The heterogeneity of regional microglia was increasingly recognized using NLRP3 (*Nlrp3*^+^) and Lysozyme (*Lyz2*^+^) labeling, which was confirmed by immunofluorescence staining to localize these 2 clusters of microglia around the injured area. For example, proliferative zone–associated microglia have been found in developing white matter and white matter–associated microglia in aged white matter (29, 30). In Alzheimer’s disease, DAMs surround amyloid plaques and persistently phagocytose amyloid-β aggregates (31). Understanding the regional heterogeneity of microglia and their functions may provide important clues for developing new therapies for different neurological diseases. Recent reports have shown that the M2 markers *Mrc1* (encoding CD206) and *Arg1* are predominantly expressed in borderline-associated macrophages, but not in microglia (13, 32). Furthermore, the M1/M2 paradigm focuses only on inflammation, neglecting other important biological functions and the spatial heterogeneity of microglia. Our proposed *Nlrp3*^+^ and *Lyz2*^+^ microglia further negate the M1/M2 paradigm for microglia after TBI.

Microglia stimulated by different microenvironments also showed different metabolic profiles. Several metabolic pathways related to amino acids and ketone bodies were enriched in *Lyz2*^+^ microglia compared with the higher level of lipid metabolism in *Nlrp3*^+^ microglia. Metabolism of branched-chain amino acids is thought to be neuroprotective by blocking microglia-mediated proinflammatory responses (33, 34). Glycolysis or interruption of the TCA cycle promotes a proinflammatory response in microglia, whereas an intact TCA cycle and OXPHOS maintain microglia dynamic homeostasis and inhibit excessive inflammation. In this study, aging significantly promoted glycolysis and inhibited TCA in microglia. In contrast, when *ELF1*, a key TF in *Nlrp3*^+^ microglia, was knocked down, glycolysis was inhibited and TCA cycling was restored to ensure energy supply.

Epigenetic modifications can regulate gene expression without altering the genomic sequence (35, 36). The human epigenome is extremely dynamic and constantly changing in response to environmental exposure and the aging process. Differences in chromatin accessibility between aged and young microglia may be an important cause of differences in microglia profiles after TBI. In this study, we connect aging-enhanced glycolysis with enhanced aging-induced inflammatory responses in microglia through ATAC-seq. Using the SCENIC algorithm and motif enrichment, combined with the in vitro CRISPR/Cas9 screening system, we identified a group of TFs (*ELF1*, *NFKB2*, *RELA*, *FLI1*, *NFKB1*, *JUN*, *JUNB*, and *FOSB*) capable of regulating the generation of *Nlrp3*^+^ microglia, with *ELF1* ablation having the greatest effect on the generation of *Nlrp3*^+^ microglia. Unfortunately, we did not identify the specific class of chromatin modifications that leads to chromatin opening, which requires subsequent experiments. Previous studies have shown that *ELF1* binds to the NIS-lncRNA promoter to upregulate CCL2 expression and thus induce pathological pain (37). In this study, ablation of *ELF1* in microglia in vivo significantly inhibited the flux of inflammation-related signaling pathways (MAPK and NF-κB signaling pathways) and reduced the transcription of proinflammatory cytokines. The inflammation-suppressing effect of *ELF1* ablation represents its broad-spectrum initiating effect on proinflammatory cytokines.

Imeglimin targets cellular mitochondrial dysfunction and is an oral hypoglycemic agent with dual mechanisms (18, 38). In this study, we determined that Imeglimin, as an inhibitor of ELF1, can cross the blood-brain barrier and thereby reduce the population of *Nlrp3*^+^ microglia after TBI. Treatment carried out at a concentration of 135 μg/kg did not cause fluctuations in blood pressure of sham or TBI mice. The results of histological sections of the lungs, liver, spleen, kidneys, and heart under different conditions of Imeglimin treatment are also not suggestive of organ toxicity, which suggests the safety of Imeglimin. Excessive elevation of blood glucose after TBI is thought to be negatively correlated with TBI prognosis; aging-induced enhancement of microglial glycolysis leads to a decrease in the ability of microglia to process glucose; and accumulation of oxygen free radicals induced by high glucose further aggravates the elevation of inflammation in microglia, which could be well ameliorated by the pharmacological effect of Imeglimin through blocking ELF1 (39–41). As an FDA-approved and marketed drug (18), Imeglimin has the potential to be used in the treatment of TBI, but more preclinical evidence remains to be provided.

A critical limitation of our study is the lack of age-matched non-TBI human brain tissues as control. This absence prevents us from definitively distinguishing between age-related baseline differences in microglial phenotypes and changes specifically induced by TBI in human subjects. For instance, we cannot fully rule out whether the higher proportion of *Nlrp3*^+^ microglia or lower abundance of Lyz2^+^ microglia observed in aged patients with TBI reflects a preexisting age-associated shift in microglial homeostasis, rather than a direct response to traumatic injury. Despite these limitations, our integrative multiomics approach and cross-species validation provide convergent evidence linking age, microglial heterogeneity, metabolic-epigenetic crosstalk, and TBI outcomes, laying the foundation for future therapeutic interventions targeting ELF1-mediated microglial dysfunction.

## Methods

### Sex as a biological variable.

Our study examined male and female animals, and similar findings are reported for both sexes.

### Human brain samples.

Human studies were conducted in accordance with the Declaration of Helsinki. Inclusion of human subjects and supporting documentation were approved by the Ethics Committee of Nantong University Affiliated Hospital (approval no. 2023-K187-01). Written informed consent of all subjects or legal representatives was obtained at enrollment. Contusion brain tissue was obtained from patients with moderate to severe TBI undergoing hematoma evacuation and decompression craniotomy. We obtained brain tissues from the contused area of patients who underwent surgery within 24 hours, which included both gray and white matter, with a tissue size of approximately 1–3 cm^3^.

Brain tissue was minced and enzymatically dissociated into single-cell suspensions using a papain-based neural tissue dissociation kit. After centrifugation in 30% Percoll to remove myelin debris, single cells were suspended in 1% BSA.

A total of 13 young (<60 years old) and 22 aged (>65 years old) subjects were included in the study. Patient characteristics are provided in Supplemental Tables 1 and 2.

### Mice.

C57BL/6J-*Elf1^em1fl^* (*Elf1^fl/fl^*), C57BL/6J-*Nlrp3^em1fl^* (*Nlrp3^fl/fl^*), and C57BL/6J-*Lyz2^em1fl^* (*Lyz2^fl/fl^*) mice were purchased from Cyagen Biosciences. C57BL/6-*Cx3cr1^em1(creERT2-WPRE-polyA)^* (*Cx3cr1*^c*reERT*2^) mice were purchased from Shanghai Model Organisms Center. B6.Cg-*Gt*(ROSA)26Sor^tm9(CAG-tdTomato)Hze^/J (Ai9 reporter mice) was provided by Yang Yang (Wuxi Taihu Hospital, Wuxi, China). Young (6–8 weeks) and aged (16–18 months) WT C57BL/6 mice were either bred and aged in house or purchased from Chengdu Dossy Experimental Animals Co., Ltd. All mice used in this study were not sex specified. Experimental mice were kept in an air-conditioned room (22°C–25°C) with standard 12 hour light/12 hour dark cycles and free access to food and water. The NIH Guide for the Care and Use of Laboratory Animals was followed for all animal experiments. The experimental procedures used in this study were approved by the Experimental Animal Ethics Committee of Nantong University (approval no. IACUC20231125-1001).

### Mice genotyping.

Tail specimens (0.2–0.5 cm in length) were harvested from 2-week-old mice using sterile surgical scissors sanitized with 70% ethanol. Genomic DNA was isolated following the standard protocol of the Beyotime Mouse Tail Genotyping Kit (D7283M). Subsequent to DNA extraction, PCR was carried out with specific primer sets, and the resulting PCR amplicons were subjected to electrophoresis on a 2% agarose gel impregnated with GelRed (Biotium) at 120 V for 30 minutes. The molecular weights of the target bands were verified by comparison with the DL2000 DNA ladder (Takara).

*Elf1^fl/fl^* (forward primer, 5′-GCTGCTGAAGATGCTGATGG-3′; reverse primer, 5′-GGATGGTGGTGAAGGTGAAG-3′) yielded a 394 bp product for floxed alleles and a 320 bp product for WT alleles. *Cx3cr1*-*Cre*^ki/wt^ (forward primer, 5′-TGGCTGCTGCTGTTGTTGTA-3′; reverse primer, 5′-CAGAGACGGAAATCCATCGCT-3′) amplified a 428 bp knockin product and a 286 bp WT product.

### TBI model.

TBI was induced in mice using the controlled cortical injury (CCI) model. Briefly, anesthesia was induced and maintained in mice using isoflurane, the heads of mice were fixed in a stereotaxic frame, and a heat pack was placed under the body to maintain body temperature. A midline incision was made on the scalp, and a 2 mm diameter bone window was created 2.0 mm lateral to the median sagittal line and 1.0 mm posterior to the bregma. The brain was tilted at a 15° angle and perpendicular to the impactor (4 mm diameter tip; RWD Life Sciences). The impactor parameters for CCI were as follows: impact velocity, 3.5 m/s; deformation depth, 1.30 mm; and duration, 400 ms. After CCI, the skin incision was sutured and treated with antibiotic ointment to prevent infection. Finally, the mice were placed on a heating pad to maintain core body temperature until they recovered from anesthesia. For the sham group, craniotomy was performed and the dura was exposed, but no impact was performed.

### rmTBI model.

The rmTBI model was established as described previously (42). Briefly, mice were induced and maintained under anesthesia using isoflurane. The heads of the mice were shaved, and the heads were immobilized in the stereotaxic apparatus. A heating pad was placed under the torso to maintain body temperature, and noninvasive rubber pads were fixed on both sides of the head to prevent lateral displacement during impact. The tip of a 5 mm blunt-ended metal impactor was positioned above the sagittal suture before each impact. Impacts were applied to the intact skulls at an impact velocity of 5 m/s, a strike depth of 1.0 mm, and a dwell time of 200 ms, a condition in which the instantaneous impact force applied to the head of the mice was approximately 72 N. All mice experienced transient apnea (<20 seconds), and no skull fractures were observed. After surgery, the mice were placed on a heating pad until they regained voluntary movement and were subsequently returned to their cages and provided with soft chow and water. rmTBI group mice were subjected to the injury procedure at a frequency of 1 per day at 24-hour intervals (5 times per week, Monday–Friday), for a cumulative total of 20 impacts. To control for repeated anesthetic effects, mice in the sham-operated group received the same anesthetic regimen but were not subjected to the impingement procedure. The health status and behavioral abnormalities of the mice were monitored daily throughout the injury protocol and in the postoperative period.

### In vitro TBI model.

The in vitro TBI model was as previously described (43). Briefly, SH-SY5Y human neuroblastoma cells were differentiated into neurons using combined retinoic acid and brain-derived neurotrophic factor induction according to established protocols (44), followed by needle scratch injury manipulation. Simultaneously, microglia (2 × 10^5^ cells per insert of 12-well inserts) were cultured in the transwell upper permeable insert (3 mm diameter pores, Corning, REF3402). The original DMEM/F12 media (with 10% FBS) for the microglia was gradually replaced with a mixture of DMEM and Ham’s F12 supplemented with 10% FBS and 1% penicillin/streptomycin with the ratio changed slowly (50:50, 25:75, and 0:100) to allow the cells to acclimate to the difference in nutrient content and remain in a relative resting condition. After 48 hours, the microglia-containing inserts were transferred to the upper chambers of neuron culture plates, subjected to needle scratch injury, and activated for 24 hours. Subsequent procedures were then performed.

### Flow cytometry.

Mice were euthanized using isoflurane lethal anesthesia, and the brains were removed. The brains were minced and enzymatically digested into a single-cell suspension using a papain-based nerve tissue dissociation kit (Miltenyi Biotec). Myelin fragments were subsequently removed by centrifugation in 30% Percoll, and single cells were suspended in 1% BSA for antibody staining. For human samples, antibody staining was performed after obtaining single-cell suspensions as described previously. Microglia were sorted by flow cytometry after staining with anti-CD11b and CD45 antibodies. The purity of microglia (CD11b^+^ CD45^int^TMEM119^+^) was about 99% assessed by flow cytometry. The antibodies used are listed in Supplemental Table 3. Data were collected with a BD LSRFortessa SORP flow cytometer (BD Biosciences) and analyzed using FlowJo software.

### Total RNA extraction and RT-qPCR.

Microglia from the contused brain tissue of TBI patients or the injured cerebral hemisphere of TBI mice were sorted according to the flow cytometry gating strategy described above. Total RNA was extracted from brain tissue or microglia using the FastPure Cell/Tissue Total RNA Isolation Kit V2 (Vazyme Biotech Co., Ltd.). Subsequently, reverse transcription of 1,000 ng of extracted total RNA was performed using a reverse transcription kit (Thermo Fisher Scientific). Tissue and cell cDNAs were diluted 20- and 10-fold, respectively, in preparation for subsequent assays. RT-qPCR was performed using the Q5 system (Thermo Fisher Scientific), and *ACTB* (*Actb*) was used as an internal reference for mRNA. The PCR primer sequences are provided in Supplemental Table 4. Analysis was performed using the 2^–ΔΔCt^ method.

### Bulk RNA-seq.

Total RNA was extracted from purified microglia isolated from the ipsilateral cerebral hemisphere 3 days after TBI using the RNeasy Mini Kit (Qiagen) following the manufacturer’s instructions. The concentration and purity of the RNA were assessed using a NanoDrop 2000 spectrophotometer (Thermo Fisher Scientific), and the integrity was evaluated with an Agilent 2100 Bioanalyzer (Agilent Technologies). Only RNA samples with an RNA integrity number greater than 7.0 were used for library preparation. Libraries were prepared with Illumina TruSeq RNA Library Prep Kit v2. After library quality control and Qubit quantification (Thermo Fisher Scientific), RNA-seq was carried out on an Illumina NextSeq 2000 with paired ends. FASTQ files were mapped to the mm10 (GRCm38.p6) mouse genome, and gene counts were obtained with STAR version 2.7.2b (https://github.com/alexdobin/STAR/releases/tag/2.7.2b; commit ID b419d31). Differential gene expression analysis was performed using R package DESeq2.

### Immunofluorescence staining.

Following euthanasia of mice with isoflurane overdose, cardiac perfusion was sequentially performed using cold PBS for blood clearance, followed by 4% paraformaldehyde (PFA) perfusion for tissue fixation. After brain extraction, postfixation was conducted with 4% PFA, followed by dehydration in 30% sucrose and embedding in OCT compound (Tissue-Tek) for storage at –20°C until further processing. For contused brain tissues from TBI patients, surgical specimens were immediately immersed in 4% PFA for fixation and subjected to dehydration and embedding procedures as described above.

Human and mice brain tissues were sectioned into 10 μm thick slices using a microtome (Leica) and then were used for immunofluorescence staining. Five sections per tissue and perilesional site were selected. Antigen retrieval was performed using Quick Antigen Retrieval Solution for Frozen Sections (Beyotime, P0090) for 30 minutes. After PBS rinsing, sections were blocked with 5% normal donkey serum (Jackson Nutrition) in PBS for 2 hours, followed by staining and overnight incubation at 4°C. After secondary antibody incubation, nuclei were counterstained with DAPI (Solarbio, C0060), and slides were mounted using antifade mounting medium (Solarbio, S2100). Immunofluorescence images of mouse injured hemispheres or human brain tissues were acquired at ×10 or ×20 magnification using the navigated imaging function of a Leica Thunder 3D Assay inverted fluorescence microscope, with subsequent panoramic tissue reconstruction performed via LASX software. Fiji (ImageJ, NIH) software was employed for image processing and quantitative analysis of the perilesional cortex, including calculations of cellular coverage area and cell counts.

### Intracerebroventricular viral injection.

Adeno-associated viral vectors (rAAV-m*Cx3cr1*-*Cre*-WPRE-SV40pA [AAV-*Cx3cr1*-*Cre*] and rAAV-m*Cx3cr1*-WPRE-SV40pA [AAV-*Cx3cr1*], AAV2/MG1.2, 2.00 × 10^12^ vg/mL) (PackGene Biotech) were injected into the right cortex (point 1: 1.7 mm posterior, 1 mm lateral [right], and 1.0 mm ventral relative to bregma; point 2: 1.7 mm posterior, 2 mm lateral [right], and 1.0 mm ventral relative to bregma). The injection volume was 0.5 μL for each point at a rate of 0.25 μL/min, and the needle was left in the brain for 5 minutes after injection to prevent leakage. The burr hole was closed with bone wax, and the incision was closed with sutures. Mice were placed in separate recovery cages. TBI models were established after the viral growth reached its peak on day 28.

### Cell culture.

SH-SY5Y human neuroblastoma cell line and HMC3 were purchased from BeNa Culture Collection, and cells were cultured in DMEM/F12 containing 10% FBS and 1% penicillin/streptomycin in an incubator at 37°C/5% CO_2_.

### Seahorse assays.

Microglia were collected from mice brain tissue by FACS. Purified microglia were cultured in DMEM/F12 containing 10% FBS and 1% penicillin/streptomycin in an incubator at 37°C/5% CO_2_. Cultured plates were used for 5 × 10^5^ microglia sorted from mice brains or from different groups of HMC3 cell lines. ECAR and OCR were measured using an XF24 Seahorse Extracellular Flux Analyzer following the manufacturer’s instructions (Agilent Technologies). In the seahorse assays, microglia were treated with glucose (10 mM), oligomycin (1.5 μM), and 2-deoxy-d-glucose (MedChemExpress, HY-13966; 50 mM) for ECAR, and oligomycin (0.25 mM), FCCP (0.25 mM), rotenone (0.25 mM), and antimycin A (0.25 mM) for OCR. Each condition was performed with 3 replicates.

### Evaluation of neurological deficit.

Neurological function assessment was independently performed by 2 investigators who were blinded to treatment assignment. Neurological deficits in mice were assessed using mNSS, which comprehensively evaluates motor, sensory, reflex, and balance functions through a battery of tests. Detailed scoring information is provided in Supplemental Table 5. Scores for mice ranged from 0 to 18; 13–18 indicate severe injury, 7–12 indicate moderate injury, and 1–6 indicate mild injury.

### Rotarod test.

Mice were trained on an accelerated (5–30 rpm) rotarod for 3 days. During the test phase, mice were placed on an accelerating rotary rod with the speed increasing from 5 to 30 rpm over 5 minutes. The latency of mice to fall from the rod was recorded, and each mouse was trained for 5 trials (each trial run for 5 minutes, rest for 5 minutes). The final score is the average latency of the 5 falls of the experimental mice. Rotarod testing was performed 30 days after TBI. All experiments and data analyses were performed in a blinded manner.

### Virus infection.

The construction of vectors and retroviral packaging were performed by Packgene Biotech. Virus overexpressing *ELF1* was purchased from Packgene Biotech. First, Cas9 protein fused with GFP tag was transfected into HMC3 cells using lentivirus. Then, the successfully transfected HMC3-Cas9-GFP cells were mixed with HMC3 cells at a ratio of 3:7. In the presence of 8 μg/mL polybrene (Beyotime, C0351), the retrovirus was transduced at 1260*g* for 90 minutes at 32°C to infect HMC3 cells. After 6 hours, the culture medium was removed and replaced with fresh culture medium. Three sgRNA plasmids were constructed for each target gene, and the sgRNA with the highest editing efficiency and editing rate of more than 50% for each target gene was selected for screening. The screened sgRNA sequences and editing efficiency in HMC3 cells are listed in Supplemental Table 6.

### Compound screening for ELF1.

The 3D structure of human elf1 was downloaded from the AlphaFold website (AlphaFold ID AF-P32519-F1). The DNA binding domain was intercepted and the protein was hydrogenated using the Protein Preparation Wizard module. This was followed by energy optimization (OPLS2005 force field, RMSD of 0.30 Å). The processed proteins were used to create a grid file using the Receptor Grid Generation module to generate a grid file centered on the key amino acids Leu268, Val286, and Tyr287, with the box size set to 20 Å × 20 Å × 20 Å. The 2D formats of HY-L022P FDA-approved Drug Library Plus (containing 3,230 compounds) and HY-L035P Drug Repurposing Compound Library Plus (containing 5,331 compounds) were processed through the LigPrep module of Schrödinger software for hydrogenation, energy optimization, etc., and the 3D structures were outputted for virtual screening.

Virtual screening was carried out using the Virtual Screening Workflow module. The prepared compounds were imported, and molecular docking was performed using the Glide module. Molecular docking refers to the mutual docking between receptor and ligand molecules through geometric and energy matching. First, the standard (SP) mode in the Glide module was used to screen the prepared small-molecule compounds from HY-L035 Drug Repurposing Compound Library Plus (MedChemExpress). The top 10% of small-molecule compounds with the highest scoring values were selected, and a second round of screening using the high-precision (XP) mode was conducted to obtain the ranking of the small-molecule compounds.

Finally, we calculated the possibility of crossing the blood-brain barrier based on the lipid solubility and molecular weight of the compounds. The top 10 compounds were selected for validation based on the absolute value of docking score.

### Drug administration.

Tamoxifen (MedChemExpress, HY-13757A) was dissolved in ethanol/corn oil (1:9) at a concentration of 30 mg/mL, and Cx3cr1^creERT2/wt^ *Elf1*^fl/fl^ mice received 100 μL (3 mg per mouse) daily for 3 consecutive days by intraperitoneal injection. Imeglimin (MedChemExpress, HY-14771A) was dissolved in 10% DMSO and 90% corn oil. Mice received intraperitoneal injections of 150 μg/kg/day Imeglimin or equal volumes of solvent. Injections of Imeglimin or vehicle were started on the day after TBI and continued for 7 consecutive days. MCC950 was purchased from MedChemExpress (HY-12815).

### Detection of cytokine concentrations and blood glucose in serum.

Serum levels of IL-6 and IL-1β in mice were measured using the Mouse IL-6 ELISA Kit (Multi Sciences, EK206) and the Mouse IL-1β ELISA Kit (Multi Sciences, EK201B) according to the manufacturer’s instructions.

Serum levels of glucose in mice were measured using the a glucose fluorometric assay kit (Multi Sciences, E-BC-F037) according to the manufacturer’s instructions.

### Measurement of mice blood pressure.

Blood pressure was assessed in mice using the BP-2000 Blood Pressure Analysis System (Visitech Systems) through tail-cuff transmission photoplethysmography. Briefly, mice were placed in a restraint device allowing tail passage through a cuff equipped with optical sensors. The standard test session was run 20 times, and systolic blood pressure, diastolic blood pressure, and heart rate parameters were acquired.

### Preparation of single-cell suspensions from mouse cerebral hemispheres.

Fresh mouse cerebral hemispheres were rinsed in sterile PBS to remove blood and debris. The tissue was then finely minced with sterile scissors and forceps to increase the surface area for enzymatic digestion. The minced tissue was digested enzymatically by incubating it with 0.05% Liberase TL (Roche) in PBS at 37°C with gentle shaking for 15–30 minutes. After digestion, the tissue was mechanically dissociated by passing it through a 40 μm sterile nylon mesh to generate a single-cell suspension. This suspension was centrifuged at 300*g* for 5 minutes to pellet the cells, which were subsequently resuspended in sterile PBS or culture medium. The cell suspension was then filtered through a 40 μm cell strainer to remove any remaining debris or clumps. Cells were counted using a hemocytometer or automated cell counter, and viability was assessed with trypan blue staining. The resulting single-cell suspension was either used immediately for downstream applications, stored at 4°C for short-term use, or frozen with a cryoprotectant for long-term storage.

### scRNA-seq library preparation and sequencing.

scRNA-seq was conducted by Shanghai GeneChem Co., Ltd. In brief, cell suspensions were processed to generate scRNA-seq libraries using the Chromium Next GEM Single Cell 5′ GEM, Library, and Gel Bead Kit v3.1 (10x Genomics) according to the manufacturer’s protocol, as outlined below. Briefly, cell samples (16,500 cells per sample) were loaded into a Chromium Single-Cell Instrument (10x Genomics) to generate single-cell gel bead-in-emulsions (GEMs), targeting the capture of 10,000 cells per sample. Reverse transcription within GEMs (GEM-RT) was performed to synthesize barcoded full-length cDNA from polyadenylated mRNA. After GEM disruption, the GEM-RT reaction mixtures were pooled, and cDNA was purified using silane magnetic beads (Thermo Fisher Scientific, DynaBeads MyOne Silane Beads). The purified cDNA was then amplified by PCR, followed by enzymatic fragmentation and size selection to optimize cDNA amplicon size. Indexed sequencing libraries were constructed through end repair, A-tailing, adaptor ligation, and PCR amplification. The final libraries, containing P5 and P7 priming sites for Illumina bridge amplification, were sequenced on an Illumina NovaSeq 6000.

### scRNA-seq data analysis.

All scRNA-seq analysis was performed in R (version 4.0.2) using the Seurat package (version 4.1.1). Cells with fewer than 200 detected genes, more than 5,000 detected genes, or over 10% mitochondrial gene content were filtered out to remove low-quality or apoptotic cells. Data normalization was performed using Seurat’s NormalizeData function, employing a log-normalization method to account for differences in sequencing depth. The FindVariableFeatures function was used to identify highly variable genes, followed by data scaling with the ScaleData function, which regresses out unwanted sources of variation such as total unique molecular identifier counts and mitochondrial content. We applied the Harmony algorithm using the RunHarmony function to integrate samples. Dimensionality reduction was carried out using principal component analysis through the RunPCA function, which reduced the dataset’s dimensionality while retaining key variation. For clustering, the shared nearest neighbor modularity optimization-based algorithm was applied using the FindNeighbors and FindClusters functions, typically with a resolution parameter tuned to optimize cluster granularity. Clusters were visualized using UMAP via the RunUMAP function, providing a 2D representation of the cellular landscape. Differential expression analysis was performed with the FindMarkers function, which typically uses Wilcoxon’s rank-sum test to identify genes that are differentially expressed between clusters. The SCENIC TF inference was performed using the package SCENIC (v1.2.4) following the recommended guidelines. The AddModuleScore function in Seurat was utilized to calculate the scores for the genes of interest. GSVA was employed to assess the enrichment of various pathways across different cell populations by transforming the gene expression matrix into a gene set expression matrix for these populations. Based on the pathway activity scores obtained for each cell through GSVA, differential tests were conducted to compare the pathway activity scores of each cell group against those of all other cell groups. In addition, the marker panel used to distinguish microglia from macrophages is listed in Supplemental Table 7.

### SCENIC analysis.

We employed SCENIC (v1.2.4) to delineate the TFs regulating microglia population formation. SCENIC identifies regulons, comprising TFs and their potential target genes, for each cell population and quantifies their activity. The regulon activity level serves as an indicator of the TF’s influence within the cell, with elevated activity reflecting a stronger regulatory impact. The SCENIC workflow involved running the following steps: runSCENIC_1_coexNetwork2modules to infer the gene regulatory network, runSCENIC_2_createRegulons to predict regulons using RcisTarget databases, runSCENIC_3_scoreCells to score regulon activities, and runSCENIC_4_aucell_binarize to cluster cells based on gene regulatory network activity.

### Metabolomics liquid chromatography–mass spectrometry analysis.

Metabolomics liquid chromatography–mass spectrometry analysis was conducted by Bionovogene Co., Ltd. In brief, The ExionLC ultra-high performance liquid chromatography system (AB Sciex) was operated on an Agilent Eclipse XDB-C18 column (2.1 × 150 mm, 5 μm) with an injection volume of 5 μL, a column temperature of 40°C, and mobile phases of 6.25 mm ammonium acetate (A; containing 0.2% acetic acid) and 0.2% acetic acid (B; containing methanol). The gradient elution conditions were 0–2 minutes, 20% B; 2–6 minutes, 20%–70% B; 6–7 minutes, 70%–20% B; 7–9.00 minutes, 20% B at a flow rate of 0.4 mL/min.

Mass spectrometry analysis was performed on an AB Sciex triple quadrupole 6500 + mass spectrometer (AB Sciex) in multiple reaction monitoring mode. Electrospray ionization was operated in negative mode with the following parameters: ion spray voltage, −4,000 V; ion source temperature, 600°C; curtain gas (CUR), 30 psi; ion source gas 1 (GS1), 60 psi; ion source gas 2 (GS2), 60 psi.

### Bulk ATAC-seq.

ATAC-seq was conducted by Shanghai Jiayin Biotech. In brief, NLRP3^+^ and Lysozyme^+^ microglia were sorted from mouse brains after TBI according to the flow cytometry gating strategy. Subsequently, the hierarchically folded DNA was simultaneously cleaved using Tn5 transposase. Native nuclei were purified using the MinElute PCR Purification Kit (Qiagen, 28004) and subjected to 6 cycles of RT-qPCR amplification. As an input control, 10 ng of genomic DNA was used. For data filtering, raw reads were processed using Trimmomatic (V0.35, http://www.usadellab.org/cms/?page=trimmomatic).

BWA software (https://bio-bwa.sourceforge.net/) was used for alignment. The fragment sizes of the read pairs were calculated using the BAM files from aligned paired-end sequencing data. Summary statistics of fragment lengths were estimated by sampling several regions based on the size of the genome and the number of processors. MACS2 (V2.2.7.1, https://pypi.org/project/MACS2/) was used for peak calling in this analysis, and Bedtools (V2.30.0, https://bedtools.readthedocs.io/en/latest/) was used for peak annotation analysis. TF binding motifs were identified with the HOMER findMotifsGenome.pl tool in the chromatin-accessible region; motifs with *P* value less than 0.05 were considered significant.

### Biological function enrichment analysis.

Gene Ontology (GO) and Kyoto Encyclopedia of Genes and Genomes (KEGG) enrichment analyses were performed using the clusterProfiler R package, with an adjusted *P* < 0.05 serving as the filtering standard for functional analysis. GSEA was performed using the R package clusterProfiler, and ggpolt2 was subsequently employed to analyze and visualize GSEA data. The significance of GSEA enrichment was determined by threshold values (adjusted *P* < 0.05)

### Statistics.

The data were analyzed utilizing GraphPad Prism 10.5.0 and subsequently expressed as the mean ± SEM. We used the Shapiro-Wilk test to assess the normality of the distribution of continuous variables. All data were tested for normality, and data that did not show a normal/Gaussian distribution was analyzed by nonparametric equivalents. Comparisons were made using an unpaired 2-tailed Student’s *t* test and 1- and 2-way ANOVA. Survival analysis was performed using the Kaplan-Meier method and compared by log-rank test. A *P* value less than 0.05 was considered significant. Each experiment was replicated a minimum of 3 times.

### Study approval.

The study was approved by the Ethics Committee of the Affiliated Hospital of Nantong University (approval no. 2023-K187-01). All participants provided written informed consent. All experimental procedures were in accordance with the Guidelines on the Care and Use of Laboratory Animals and were approved by the Nantong University Laboratory Animal Ethics Committee (approval no. IACUC20231125-1001).

### Data availability.

scRNA-seq data of mice brains are available in the Genome Sequence Archive database under accession number CRA033293. All data values reported in this work are reported in the Supporting Data Values file. The article and the supplemental material present all data needed to evaluate the conclusions.

## Author contributions

Writing (original draft): Z Lu, YS, CW, Z Liu, WS, KS, and PG. Conceptualization: Z Lu, YS, Z Liu, WS, KS, and PG. Investigation: Z Lu, YS, CW, Z Liu, ZW, QL, RJ, J Zhao, WL, XZ, J Zhu, and YZ. Writing (review and editing): Z Lu, YS, CW, Z Liu, WS, KS, and PG. Methodology: Z Lu, YS, CW, Z Liu, ZW, QQL, RJ, WL, XZ, J Zhao, J Zhu, and YZ. Resources: WS, KS, and PG. Funding acquisition: WS, KS, PG, and QL. Data curation: Z Lu, YS, CW, Z Liu, ZW, QL, RJ, WL, and XZ. Validation: Z Lu, YS, CW, Z Liu, ZW, QL, RJ, WL, XZ, J Zhao, WS, KS, and PG. Supervision: WS, KS, and PG. Formal analysis: Z Lu, YS, CW, Z Liu, WS, and KS. Software: YS, Z Liu, ZW, and J Zhao. Project administration: WS, KS, and PG. Visualization: Z Lu, YS, CW, Z Liu, ZW, and J Zhao. All authors have read and agreed to the published version of the manuscript. Z Lu, YS, CW, and Z Liu contributed equally to this work.

## Conflict of interest

The authors have declared that no conflict of interest exists.

## Funding support

- National Key Research and Development Program of China (2023YFC2306502 to WS).
- National Natural Science Foundation of China (82471422 and 82271415 to PG).
- Jiangsu Province Research Hospital funding (YIXYY202204-ZD15, YJXYY202204-ZD16, and YJXYY202204-XKB12 to WS).
- National Natural Science Foundation of China (82301561 to QL, 82322023 and 82101364 to KS, and 82371398 to WS).

## Supplementary Material

Supplemental data

Unedited blot and gel images

Supporting data values

## Version 1 04/02/2026

In-Press Preview

## Version 2 06/15/2026

Electronic publication
