# Supplementary material for: Aging-dependent microglial heterogeneity worsens outcomes in models of traumatic brain injury
Source: J Clin Invest. 2026 Apr 2;136(12):e196112. doi: 10.1172/JCI196112 (PMC13262727; doi:10.1172/JCI196112)
Supplement: Supplemental data [file jci-136-196112-s296.pdf]

Supplemental Materials for

Aging-dependent microglial heterogeneity worsens outcomes in models of traumatic brain injury

Zhichao Lu[1,4,10], Yi Shuai[7,10], Chenxing Wang[1,4,10], Zongheng Liu[8,10], Ziheng Wang[5,6], Qianqian Liu[1,4], Rui Jiang[1,4], Jue Zhu[1,4], Yongqi Zhu[1,4], Weiquan Liao[1,4], Xingjia Zhu[1,4], Jingwei Zhao[9], Kaibin Shi[2,3*], Wei Shi[1,4*], Peipei Gong[1,4*]

1 Department of Neurosurgery, Research Center of Clinical Medicine, Affiliated Hospital of Nantong University, Medical School of Nantong University, Nantong, Jiangsu, China.

2 Department of Neurology, China National Clinical Research Center for Neurological Diseases, Beijing Tiantan Hospital, Capital Medical University, Beijing, China.

3 Chinese Institute for Immunology, Chinese Institutes for Medical Research, Beijing, China.

4 Neuro-Microscopy and Minimally Invasive Translational Medicine Innovation Center, Affiliated Hospital of Nantong University, Nantong, Jiangsu, China.

5 MOE Frontier Science Centre for Precision Oncology, University of Macau, Macau SAR, China.

6 The School of Public Health and Preventive Medicine, Monash University, Melbourne, Victoria, Australia.

7 Faculty of Medicine, The Chinese University of Hong Kong, Shatin, Hong Kong Special Administrative Region, China.

8 Department of Neurosurgery, Zhejiang Provincial Hospital of Chinese Medicine, The First Affiliated Hospital of Zhejiang Chinese Medical University, Hangzhou, Zhejiang, China.

9 Department of General Surgery, Xinhua Hospital, Affiliated to Shanghai Jiao Tong University

School of Medicine, Shanghai, 200092, China.

10 These authors contributed equally.

*Corresponding Authors

Peipei Gong: ntgpp@ntu.edu.cn

Wei Shi: fysw@ntu.edu.cn

Kaibin Shi: kshi@cimrbj.ac.cn

This PDF includes:

17 Supplemental Figures and 7 Supplemental Tables.

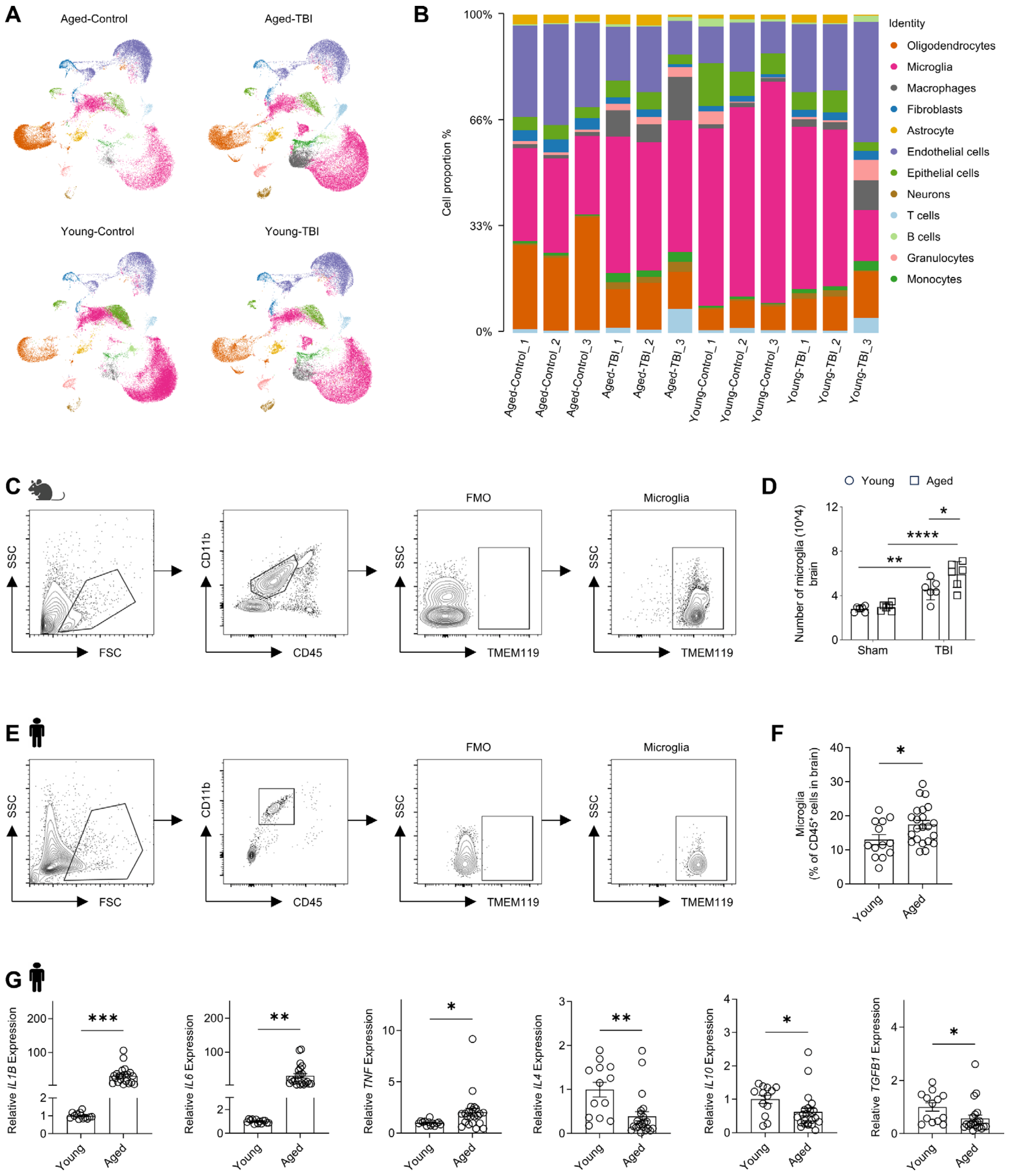

Supplemental Figure 1. Microglia responses are more intense in the aged after TBI. (A) UMAP plot shows different cell types in the brains of young sham-operated control group (Young-Ctrl), aged sham-operated control group (Aged-Ctrl), young TBI group (Young-TBI), and aged TBI group (Aged-TBI), distinguished by different colors. n=3/group. **(B)** Proportional stack plots show the percentage of different cell types in the brains from Young-Ctrl group, Aged-Ctrl group, Young-TBI group, and Aged-TBI group, distinguished by different colors. n=3/group. **(C)** Flow cytometry gating strategy for mouse microglia

(CD45^{int}CD11b^{+}TMEM119^{+}). **(D)** Bar plots show the number of microglia in the brains of young and aged mice from the sham and TBI groups. n=6/group. **(E)** Flow cytometry gating strategy for human microglia (CD45^{int}CD11b^{+}TMEM119^{+}). **(F)** Bar plots show the proportion of microglia in the brains of young and aged TBI patients. Young patients, n=13; Aged patients, n=22. **(G)** Bar plots show the transcript levels of *IL1B*, *IL6*, *TNF*, *IL4*, *IL10*, *TGFB1* expression in microglia from aged and young TBI patients. Young, n=13; Aged, n=22. Data are represented as mean ± SEM. *$P < 0.05$, **$P < 0.01$, ***$P < 0.001$, ****$P < 0.0001$. Statistical analyses were performed using two-tailed unpaired Student's t test (F, G) and two-way ANOVA followed by Tukey post hoc test (D). The schematic diagram was generated by BioRender.

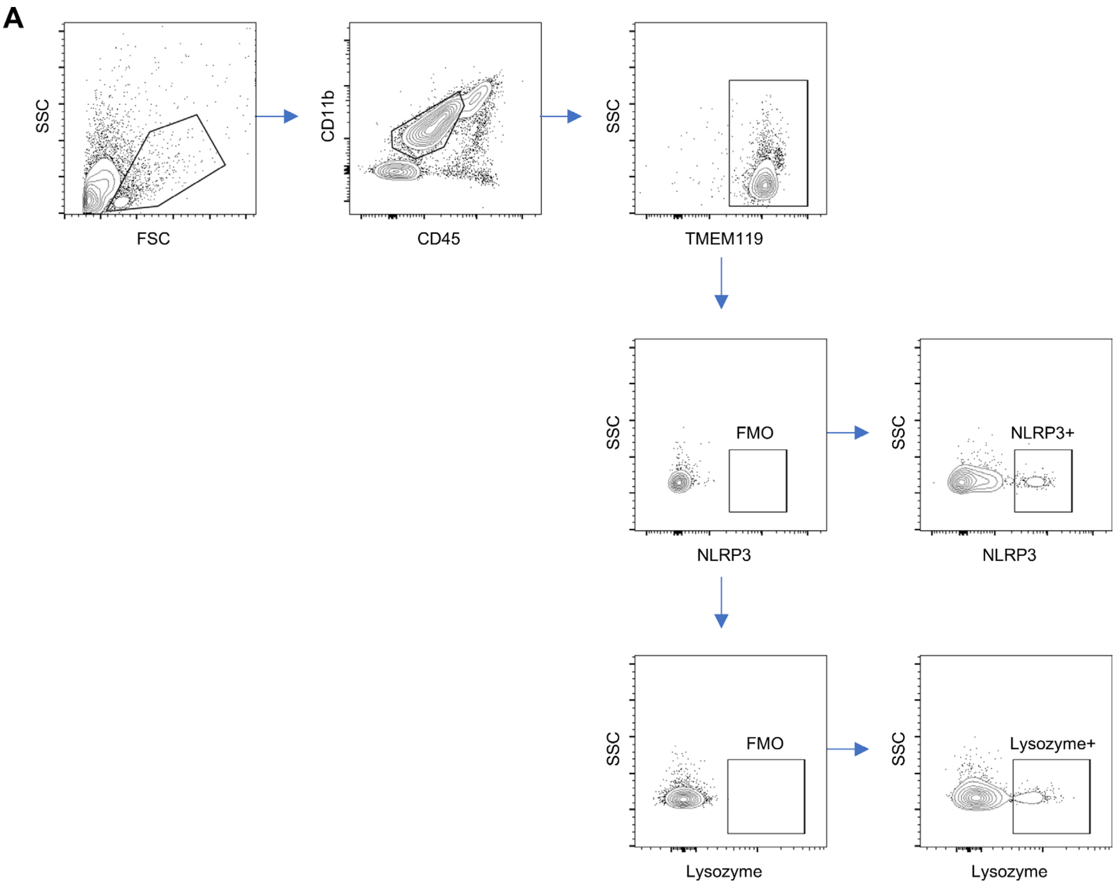

Supplemental Figure 2. Flow cytometry strategies for the detection of mouse microglia. **(A)** Flow cytometry strategies for detecting NLRP3^{+} microglia and Lysozyme^{+} microglia in mice.

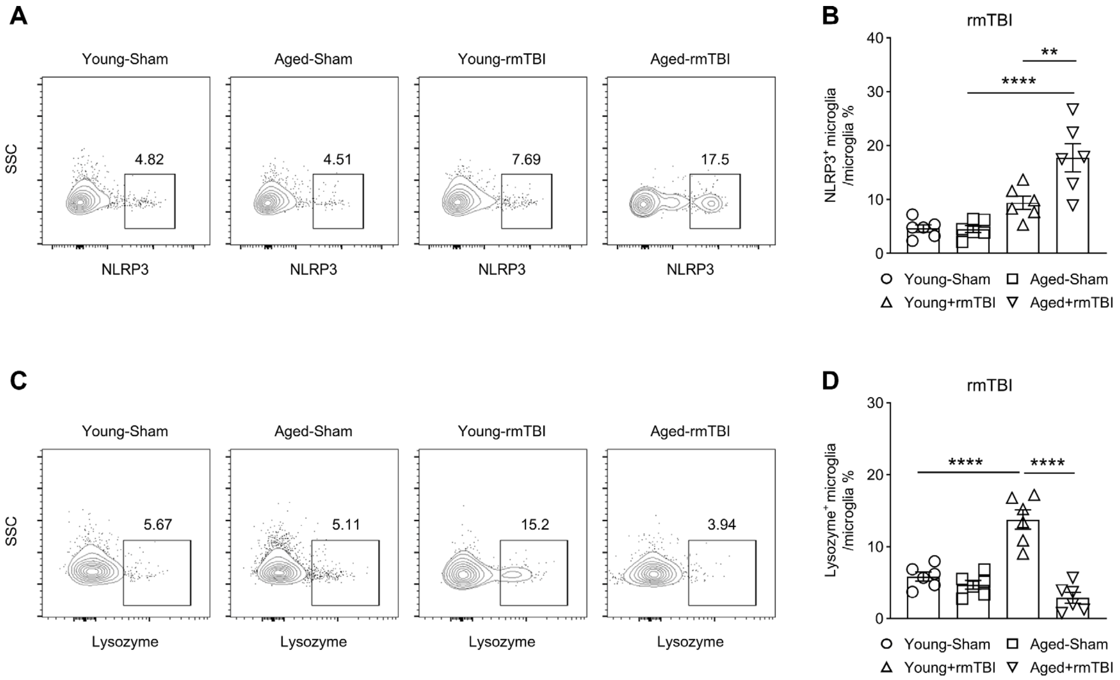

Supplemental Figure 3. Enhanced NLRP3$^+$ microglia accompanied by Lysozyme$^+$ microglia defects occurring in aged rmTBI mice. (A-B) Representative flow cytometric plots and proportion of NLRP3$^+$ microglia in the brains of repeat mild traumatic brain injury (rmTBI) mice with different treatments. n=6/group. **(C-D)** Representative flow cytometric plots and and proportion of Lysozyme$^+$ microglia in the brains of rmTBI mice with different treatments. n=6/group. Data are represented as mean ± SEM. *$P < 0.05$, **$P < 0.01$, ***$P < 0.001$, ****$P < 0.0001$. Statistical analyses were performed using two-way ANOVA followed by Tukey post hoc test (B, D).

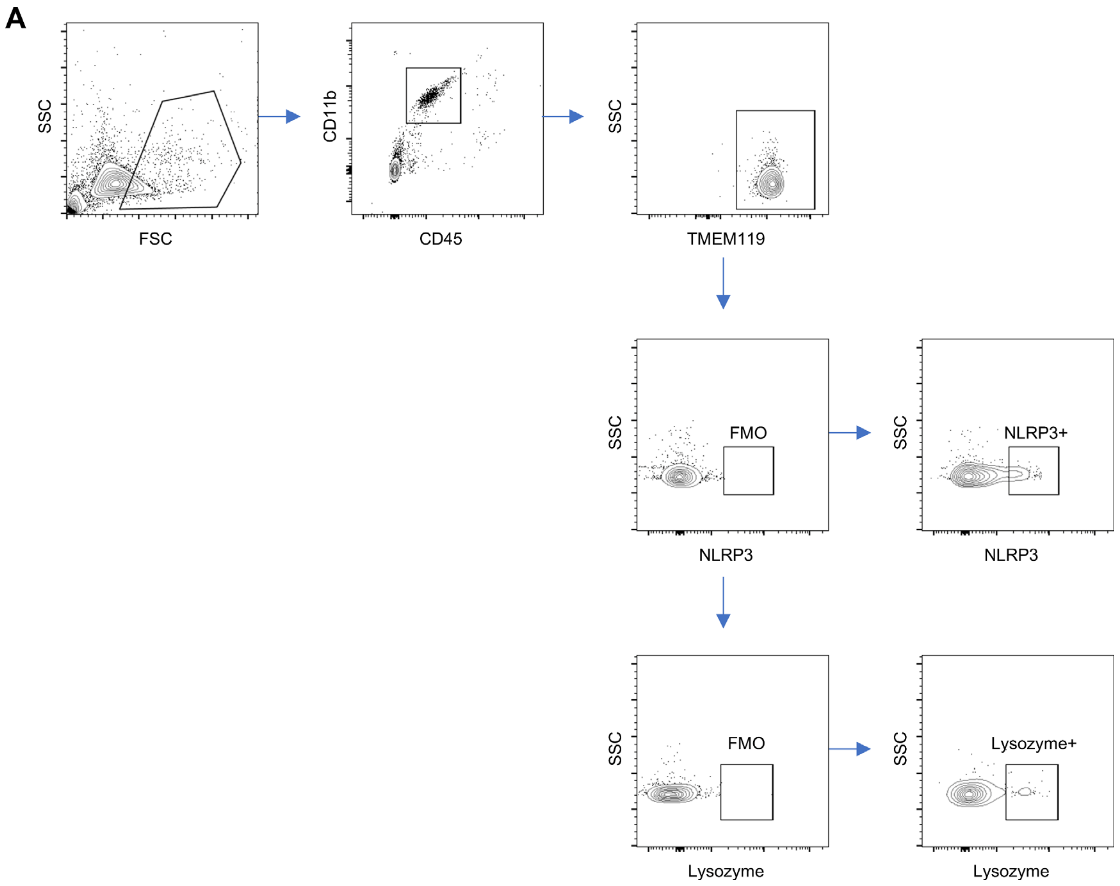

Supplemental Figure 4. Flow cytometry strategies for the detection of human microglia.

(A) Flow cytometry strategies for detecting NLRP3^{+} microglia and Lysozyme^{+} microglia in TBI patients.

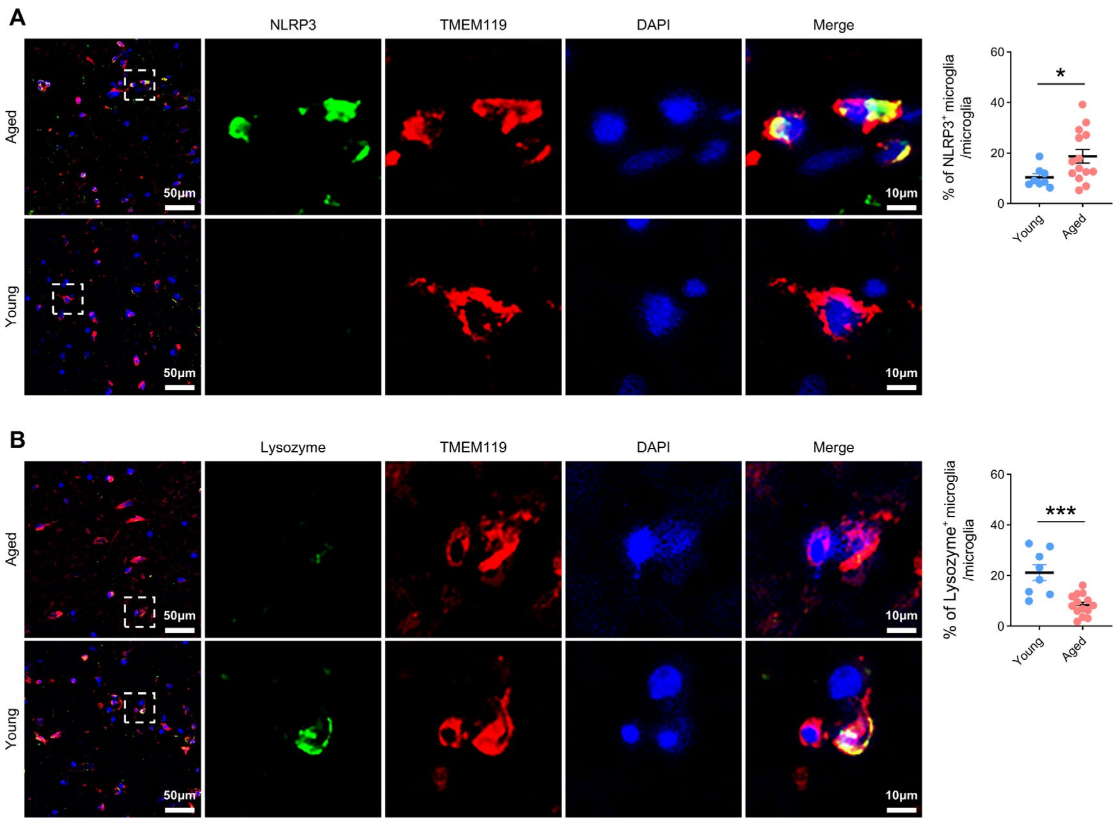

Supplemental Figure 5. NLRP3^{+} microglia accumulate in brain tissue of aged patients with TBI. (A) Representative fluorescent images of NLRP3^{+} microglia in brain tissue from aged and young TBI patients. Bar plot shows the proportion of NLRP3^{+} microglia in the field. Young, n=8; Aged, n=14. **(B)** Representative fluorescent images of Lysozyme^{+} microglia in brain tissue from aged and young TBI patients. Bar plot shows the proportion of Lysozyme^{+} microglia in the field. Young, n=8; Aged, n=14. Data are represented as mean ± SEM. **P* < 0.05, ****P* < 0.001. Statistical analyses were performed using two-tailed unpaired Student's t test (A, B).

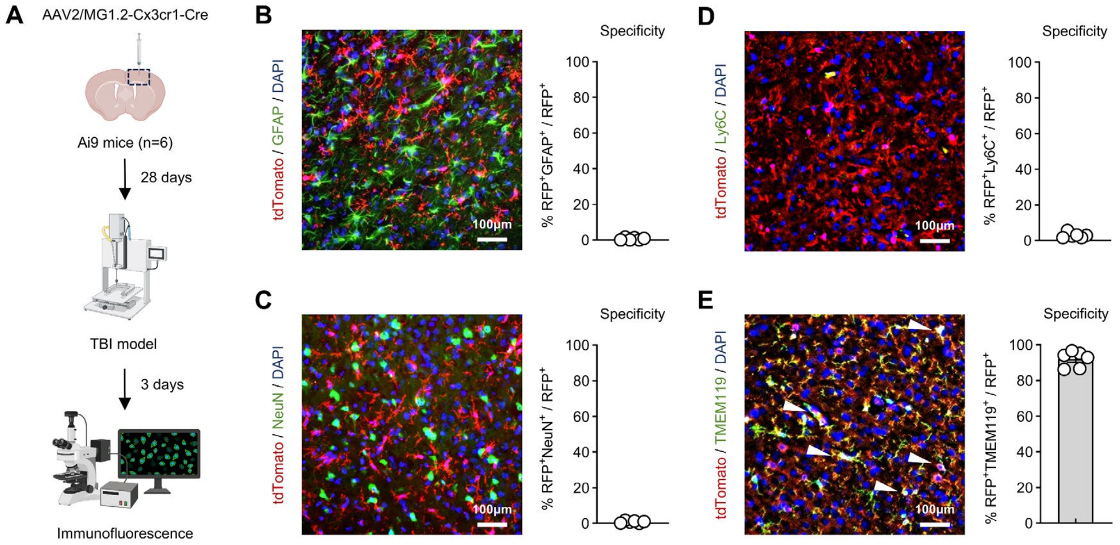

Supplemental Figure 6. Validation of the efficiency of targeting microglia. (A) Schematic of microglia-targeting strategy: AAV2/MG1.2-*Cx3cr1*-*Cre* was injected into Ai9 reporter mice's right cortex (which express tdTomato upon *Cre*-mediated recombination). TBI modeling was performed 28 days post-viral injection to ensure maximal viral transduction. The black box represents the area for collecting the fluorescent signal. **(B)** Representative immunofluorescence images showing colocalization of tdTomato (*Cre*-positive cells, red) with the astrocyte marker GFAP (green) in the injured hemisphere of Ai9 mice. Nuclei were counterstained with DAPI (blue). Scale bars: 100 μm. n=6 mice. **(C)** Representative immunofluorescence images showing colocalization of tdTomato (*Cre*-positive cells, red) with the neuron marker NeuN (green) in the injured hemisphere of Ai9 mice. Nuclei were counterstained with DAPI (blue). Scale bars: 100 μm. n=6 mice. **(D-E)** Flow cytometry analysis of tdTomato expression in monocyte (Ly6C, D), and microglia (TMEM119, E). Scale bars: 100 μm. n=6 mice. The schematic diagram was generated by BioRender.

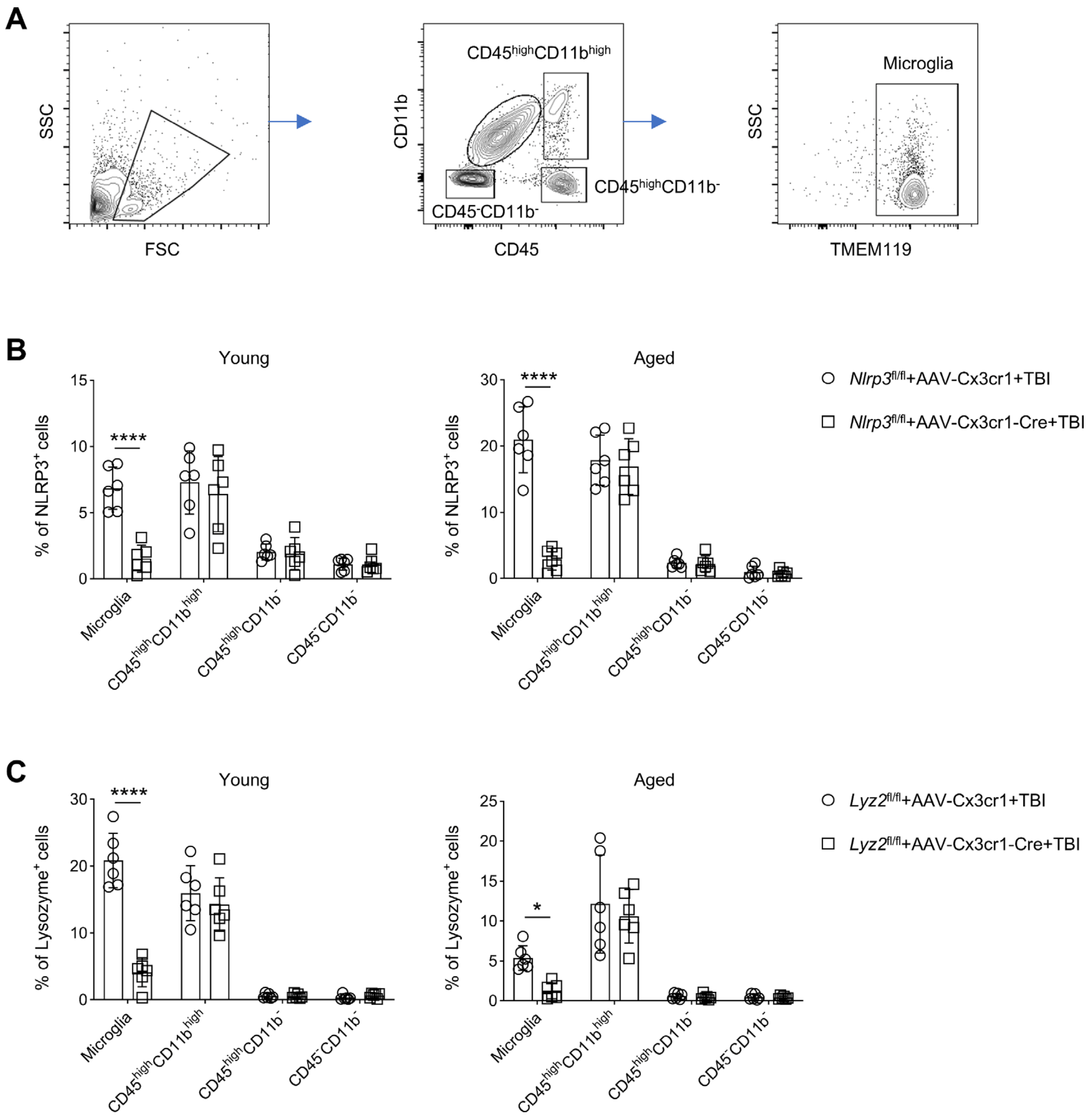

Supplemental Figure 7. Validation of the efficiency of in-vivo knockout microglial *Nlrp3* and *Lyz2*. **(A)** AAV-*Cx3cr1* or AAV-*Cx3cr1-Cre* was injected into the lateral ventricle of *Nlrp3$^{fl/fl}$* and *Lyz2$^{fl/fl}$* mice, and TBI modeling was performed 28 days later. Flow cytometry was employed to confirm the efficiency and specificity of the viral knockout strategy. **(B)** Bar plots show the proportion of NLRP3^{+} populations among microglia (CD45^{int}CD11b^{+}TMEM119^{+}), CD45^{high}CD11b^{high} cells, CD45^{high}CD11b^{-} cells, and CD45^{-}CD11b^{-} cells in the brains of young (left panel) and aged (right panel) TBI mice. n=6/group. **(C)** Bar plots show the proportion of Lysozyme^{+} populations among microglia (CD45^{int}CD11b^{+}TMEM119^{+}), CD45^{high}CD11b^{high}

cells, CD45^{high}CD11b^{-} cells, and CD45^{-}CD11b^{-} cells in the brains of young (left panel) and aged (right panel) TBI mice. n=6/group. Data are represented as mean ± SEM. *$P < 0.05$, ****$P < 0.0001$. Statistical analyses were performed using two-way ANOVA followed by Tukey post hoc test (B, C).

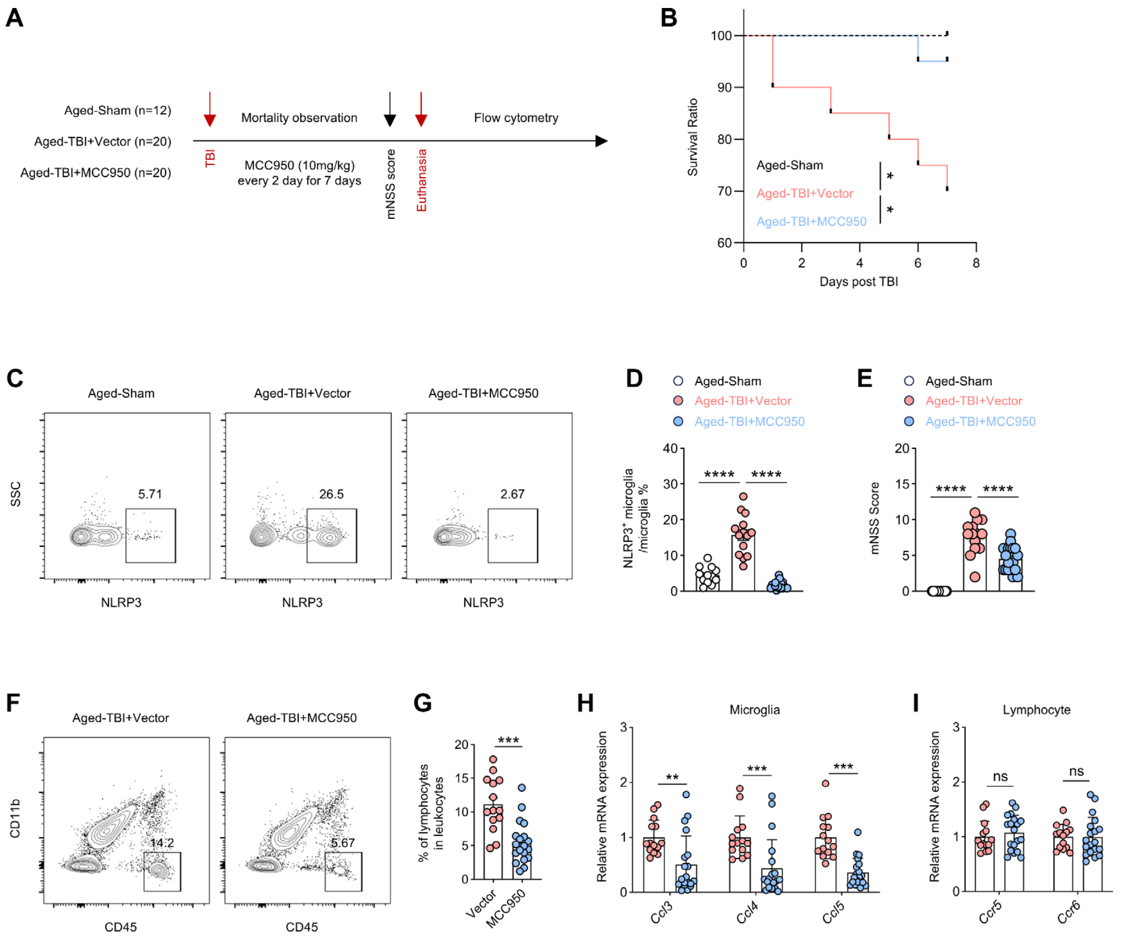

Supplemental Figure 8. MCC950 reduced mortality and NLRP3^{+} microglia response in aged TBI mice by inhibiting NLRP3. (A) Schematic of MCC950 treatment. 12 normal aged mice were used as sham controls. 40 aged mice undergoing TBI modeling were randomly and equally divided into two groups to receive vector or MCC950 treatments, respectively. Aged TBI mice received intraperitoneal injections of 10 mg/kg of MCC950 every 2 days for a total of 7 days. Mortality observation was performed during the injection phase of MCC950. The mNSS score was performed at day 7 after TBI, and the mice were euthanized and subjected to flow cytometry at the end of the score. **(B)** Survival curves of aged TBI mice receiving vector or MCC950 (10mg/kg) treatment after TBI (Start: $n_{Aged\text{-}Sham}$=12, $n_{Aged\text{-}TBI+Vector}$=20, $n_{Aged\text{-}TBI+MCC950r}$=20). **(C-D)** Representative flow cytometry results show the proportions of NLRP3^{+} microglia in aged TBI mice receiving vector or MCC950 treatment. $n_{Aged\text{-}Sham}$=12, $n_{Aged\text{-}}$

$_{\text{TBI+Vector}}$=14, $n_{\text{Aged-TBI+MCC950}}$=19. **(E)** mNSS scores of TBI under different conditions related to (B). **(F-G)** Representative flow cytometry results and bar plot show the proportion of lymphocytes in the brain following MCC950 treatment. $n_{\text{Aged-TBI+Vector}}$=14, $n_{\text{Aged-TBI+MCC950}}$=19. **(H-I)** Bar plots show the transcriptional levels of chemokines (*Ccl3*, *Ccl4*, and *Ccl5*) in microglia and chemokine receptors (*Ccr5* and *Ccr6*) in lymphocytes following MCC950 treatment after TBI. $n_{\text{Aged-TBI+Vector}}$=14, $n_{\text{Aged-TBI+MCC950}}$=19. Data are represented as mean ± SEM. *$P < 0.05$, **$P < 0.01$, ****$P < 0.001$. Statistical analyses were performed using two-tailed unpaired Student's t test (G), two-way ANOVA followed by Tukey post hoc test (D-E, H-I) and Kaplan–Meier survival analysis (B).

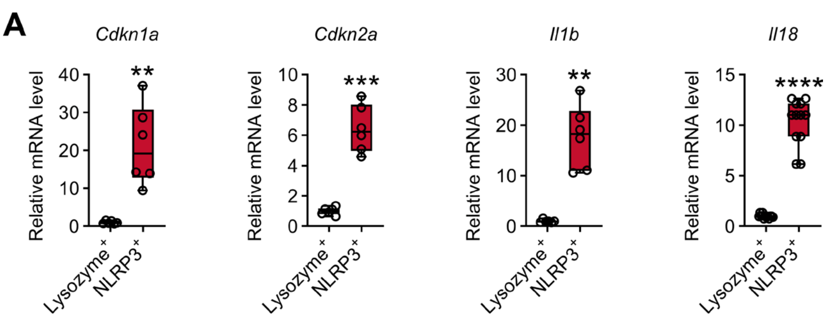

Supplemental Figure 9. NLRP3+ microglia exhibit upregulation of aging-associated markers. (A) Bar plots show the transcriptional levels of *Cdkn1a*, *Cdkn2a*, *Il1b*, and *Il18* in Lysozyme+ microglia and NLRP3+ microglia. n=6/group. Data are represented as mean ± SEM. **$P < 0.01$, ***$P < 0.001$, ****$P < 0.0001$. Statistical analyses were performed using two-tailed unpaired Student’s t test (A).

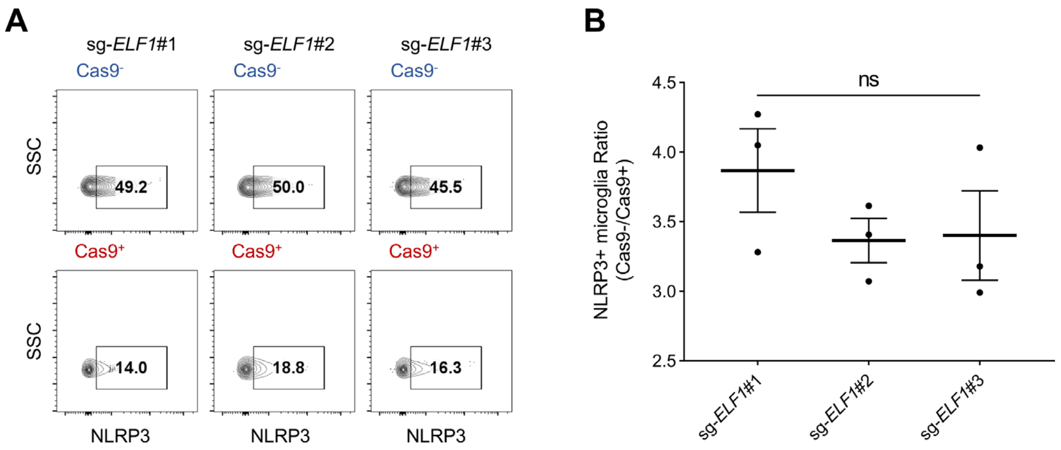

Supplemental Figure 10. Different sg-RNAs targeting *ELF1* regulate NLRP3^{+} microglia formation. (A) Representative flow cytometry results of three different sg-RNAs targeting ELF1 interventions on the fromation of NLRP3^{+} microglia. **(B)** Bar plot shows the effect of different sh-*ELF1* interventions on NLRP3^{+} microglia ratio (Cas9^{-}/Cas9^{+}). n=3/group. Data are represented as mean ± SEM. Statistical analyses were performed using one-way ANOVA followed by Tukey post hoc test (B).

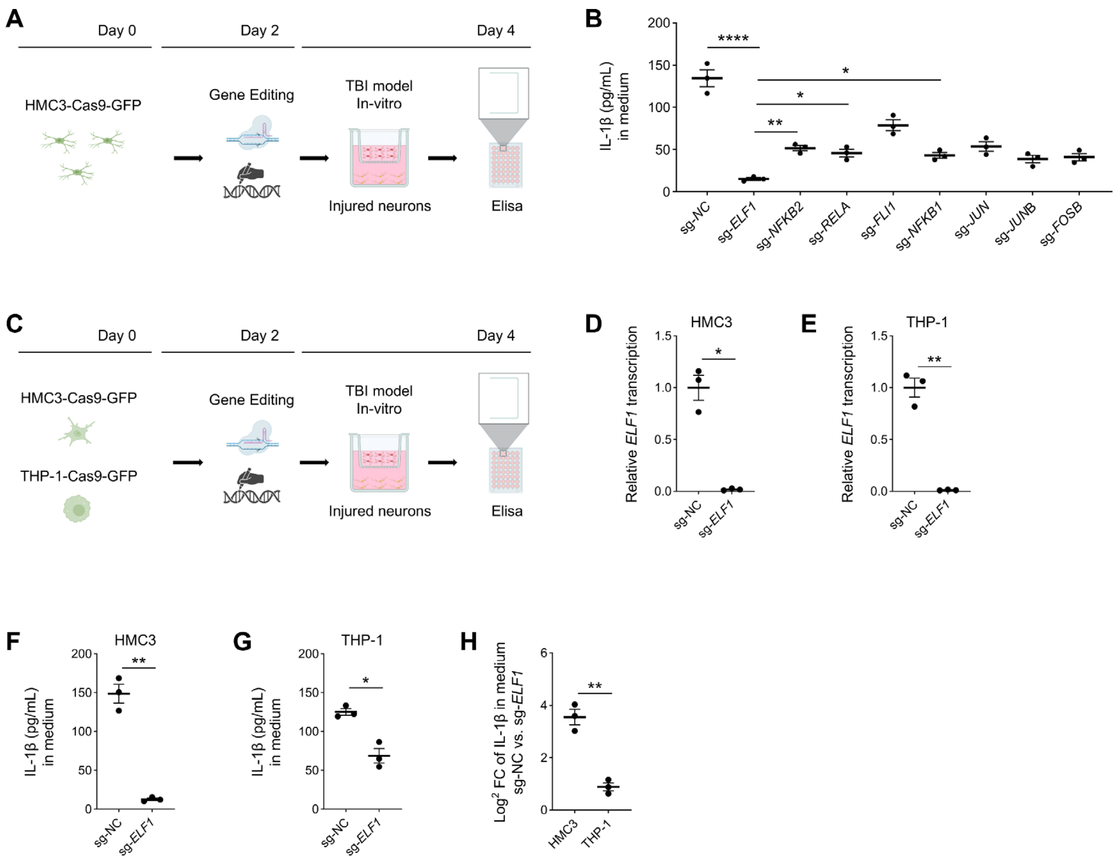

Supplemental Figure 11. In-vitro microglial *ELF1* ablation suppresses the inflammatory response. (A) Schematic diagram: we generated HMC3 microglial cell lines with deletion of different transcription factors. After in vitro TBI modeling, we compared their ability to secrete IL-1β. **(B)** Bar plots show the concentration of IL-1β in the culture medium supernatant of microglia after deletion of different transcription factors. n=3/group. **(C)** Schematic diagram: we established *ELF1* knockout HMC3 microglial cell lines and THP-1 monocyte cell lines, and compared their IL-1β release capacity. **(D)** Bar plot shows the transcription of *ELF1* in the HMC3 cell line after deletion of *ELF1*. n=3/group. **(E)** Bar plot shows the transcription of *ELF1* in the THP-1 cell line after deletion of *ELF1*. n=3/group. **(F)** Bar plot shows the concentration of IL-1β in the culture medium supernatant of HMC3 cell line after deletion of *ELF1*. n=3/group. **(G)** Bar plot shows the concentration of IL-1β in the culture medium supernatant of THP-1 cell line after deletion of *ELF1*. n=3/group. (H) Bar plot compares changes in the IL-1β secretion

capacity between wild-type (WT) HMC3 and THP-1 cell lines, and ELF1-knockout HMC3 and THP-1 cell lines. n=3/group. Data are represented as mean ± SEM. *$P < 0.05$, **$P < 0.01$, ***$P < 0.001$. Statistical analyses were performed using two-tailed unpaired Student's t test (D-H) and one-way ANOVA followed by Tukey post hoc test (B). The schematic diagram was generated by BioRender.

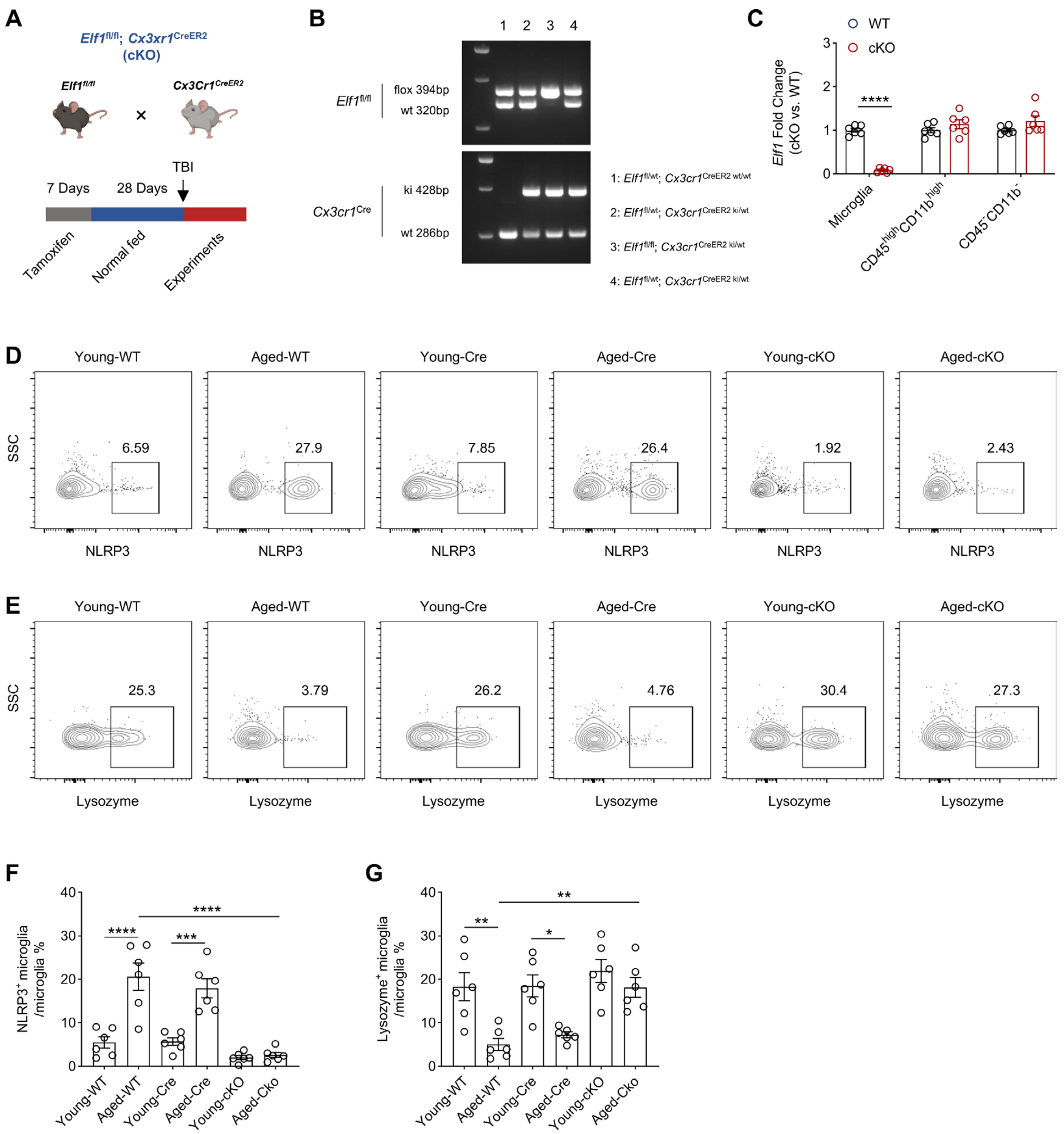

Supplemental Figure 12. Selective in-vivo knockout microglial *ELF1* decreases the proportion of NLRP3^{+} microglia after TBI. (A) Experimental strategies for selective knockout *ELF1* in microglia in vivo. **(B)** Validation of mice genotype. **(C)** qPCR validation of ELF1 expression in microglia, in macrophages and in other cells after selective knockout of *ELF1*. n=6/group. **(D-G)** Representative flow cytometry results and bar plots show that selective in-vivo knockout microglial *ELF1* decreases the proportion of NLRP3^{+} microglia (D, F) and increases the proportion of Lysozyme^{+} microglia (E, G) after TBI. n=6/group. Data are represented as mean ± SEM. **P* < 0.05, ***P* < 0.01, ****P* < 0.001, *****P* < 0.0001. Statistical

analyses were performed using two-tailed unpaired Student's t test (C) and two-way ANOVA followed by Tukey post hoc test (F-G). The schematic diagram was generated by BioRender.

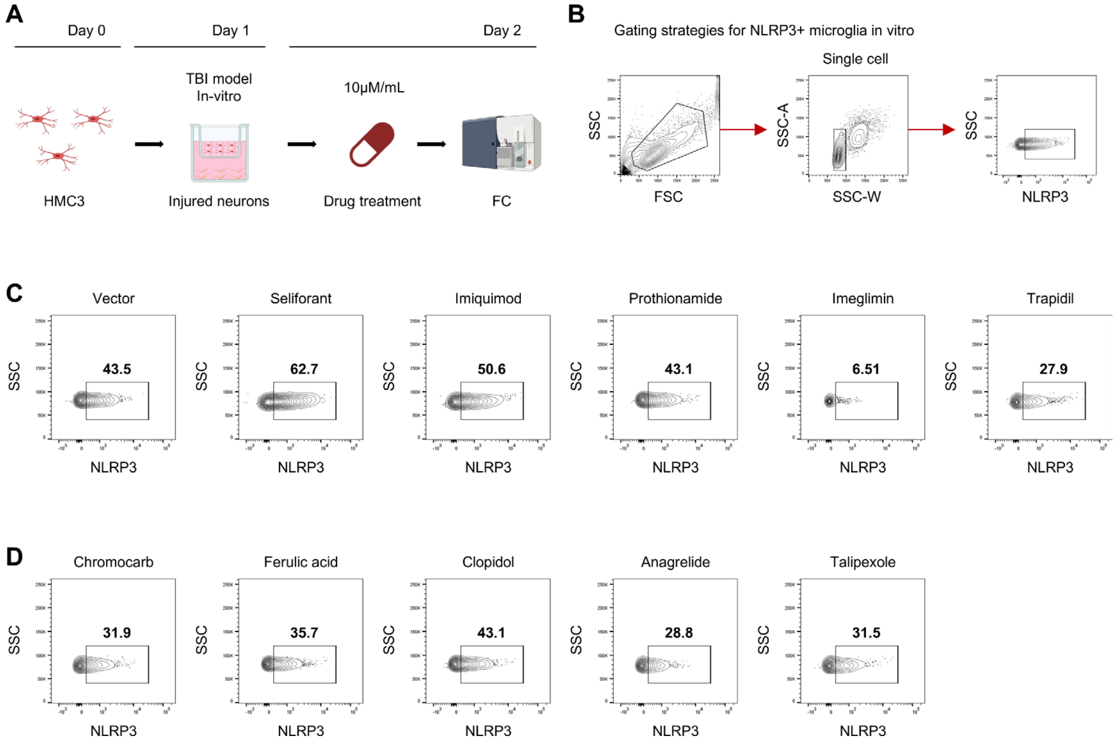

Supplemental Figure 13. Imeglimin inhibits NLRP3^{+} microglia in vitro. (A) Experimental strategy: after constructing an in vitro TBI model, the stimulated microglia were treated with different 10 μM/mL drugs and the proportion of NLRP3^{+} microglia was detected by flow cytometry. **(B)** Gating strategies for NLRP3^{+} microglia in vitro. **(C-D)** Representative flow cytometry results of NLRP3^{+} microglia after different drug treatments. The schematic diagram was generated by BioRender.

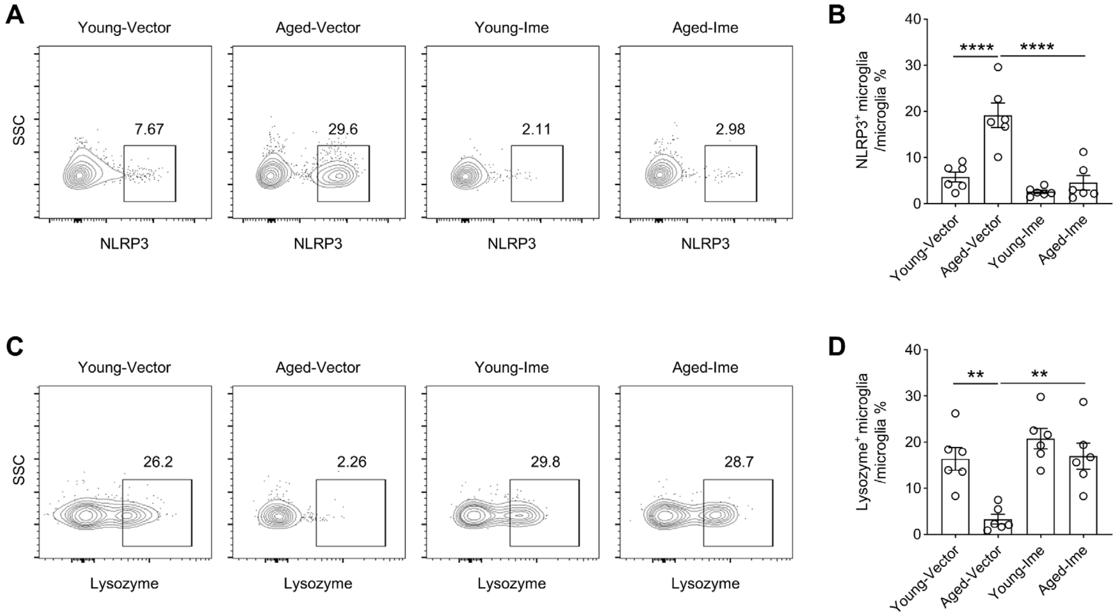

Supplemental Figure 14. Imeglimin decreases the proportion of NLRP3⁺ microglia after TBI. (A-D) Representative flow cytometry results and bar plots show that in-vivo Imeglimin treatment decreased the proportion of NLPR3⁺ microglia (A-B) and increased the proportion of Lysozyme⁺ microglia (C-D). n=6. Data are represented as mean ± SEM. *P < 0.05, **P < 0.01, ****P < 0.0001. Statistical analyses were performed using two-way ANOVA followed by Tukey post hoc test (B, D).

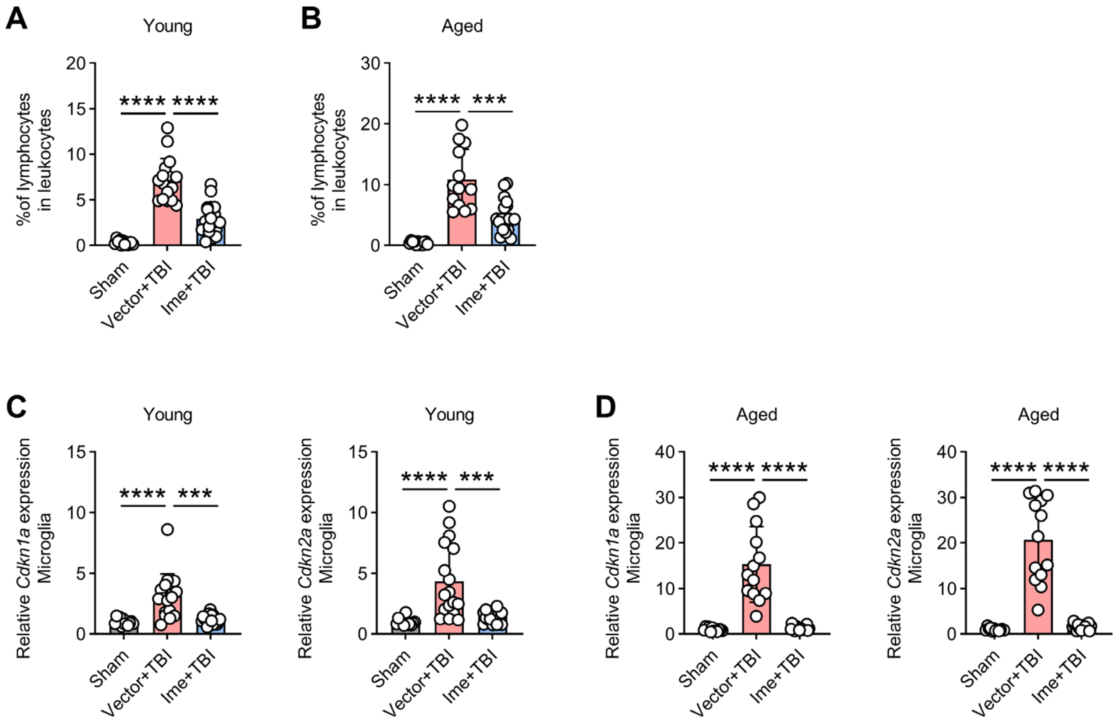

Supplemental Figure 15. Imeglimin treatment reduces lymphocyte infiltration and reverses senescence-related phenotypes of microglia following TBI. (A-B) Bar plots show the proportion of infiltrating lymphocytes in the brains of young and aged mice from three groups (Sham, Vector+TBI, and Ime+TBI) that received different treatments (Young group, n_{Sham}=12, $n_{Vector+TBI}$=17, $n_{Ime+TBI}$=18, Aged group, n_{Sham}=12, $n_{Vector+TBI}$=13, $n_{Ime+TBI}$=19). **(C-D)** Bar plots show the transcription of *Cdkn1a* and *Cdkn2a* in the microglia of young and aged mice from three groups (Sham, Vector+TBI, and Ime+TBI) that received different treatments (Young group, n_{Sham}=12, $n_{Vector+TBI}$=17, $n_{Ime+TBI}$=18, Aged group, n_{Sham}=12, $n_{Vector+TBI}$=13, $n_{Ime+TBI}$=19). Data are represented as mean ± SEM. ***P < 0.001, ****P < 0.001. Statistical analyses were performed using two-way ANOVA followed by Tukey post hoc test (A-D).

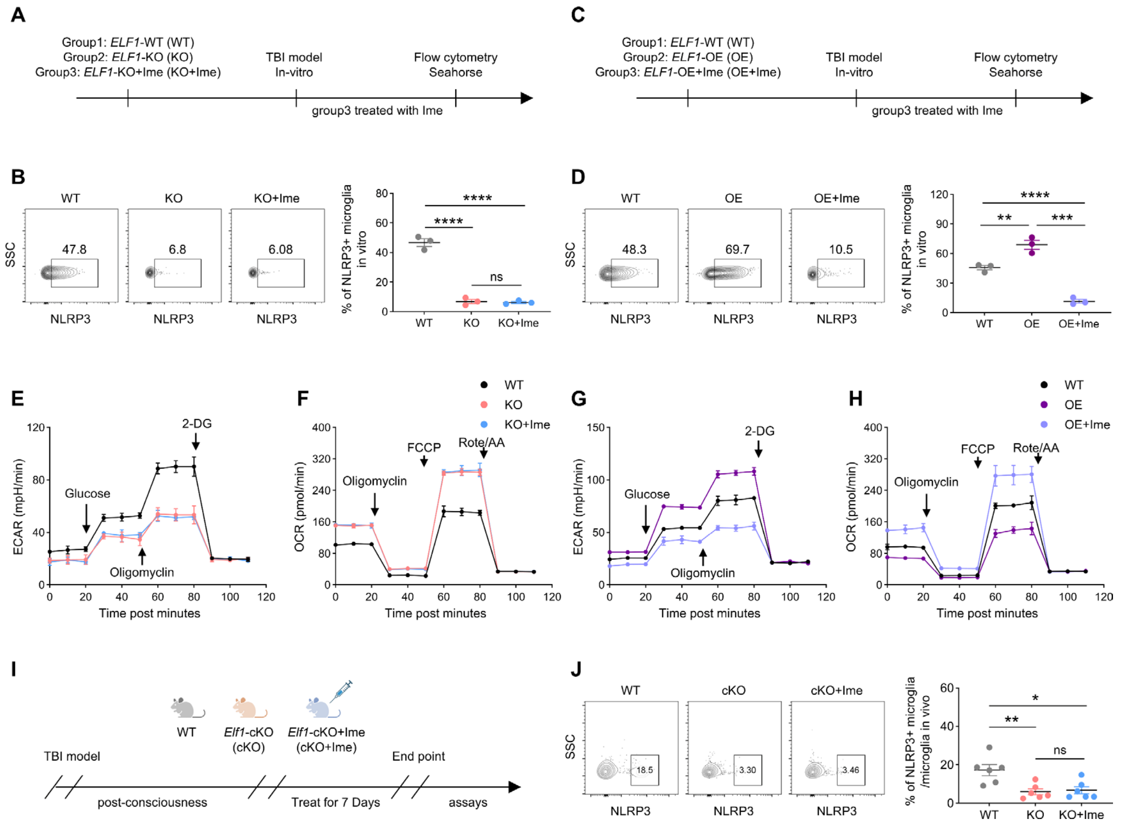

Supplemental Figure 16. Imeglimin functions through the blocking of ELF1. (A) Three groups of HMC-3 cells (WT, knockout KO, KO+Ime) were constructed, NLRP3^{+} microglia proportion and metabolic alterations were detected using flow cytometry and Seahorse. **(B)** Representative flow cytometry plots and bar plot showing the proportion of NLRP3^{+} microglia from WT group, KO group and KO+Ime group. n=3. **(C-D)** Experimental strategy and representative flow cytometry plots with bar plot show the proportion of NLRP3^{+} microglia from WT group, overexpression (OE) group and OE+Ime group. n=3. **(E-F)** ECAR and OCR results of microglia in WT, KO and KO+Ime groups. n=3. **(G-H)** ECAR and OCR results of microglia in WT, OE and OE+Ime groups. n=3. **(I)** TBI models were constructed for WT, cKO and cKO+Ime groups, and the proportion of NLRP3^{+} microglia in the brain was detected by flow cytometry. **(J)** Representative flow cytometry plots and bar plot show the proportion of NLRP3^{+} microglia from WT, cKO and cKO+Ime mice. n=6/group. Data are represented as

mean ± SEM. *P < 0.05, **P < 0.01, ***P< 0.001, ****P < 0.0001. Statistical analyses were performed using one-way ANOVA followed by Tukey post hoc test. The schematic diagram was generated by BioRender.

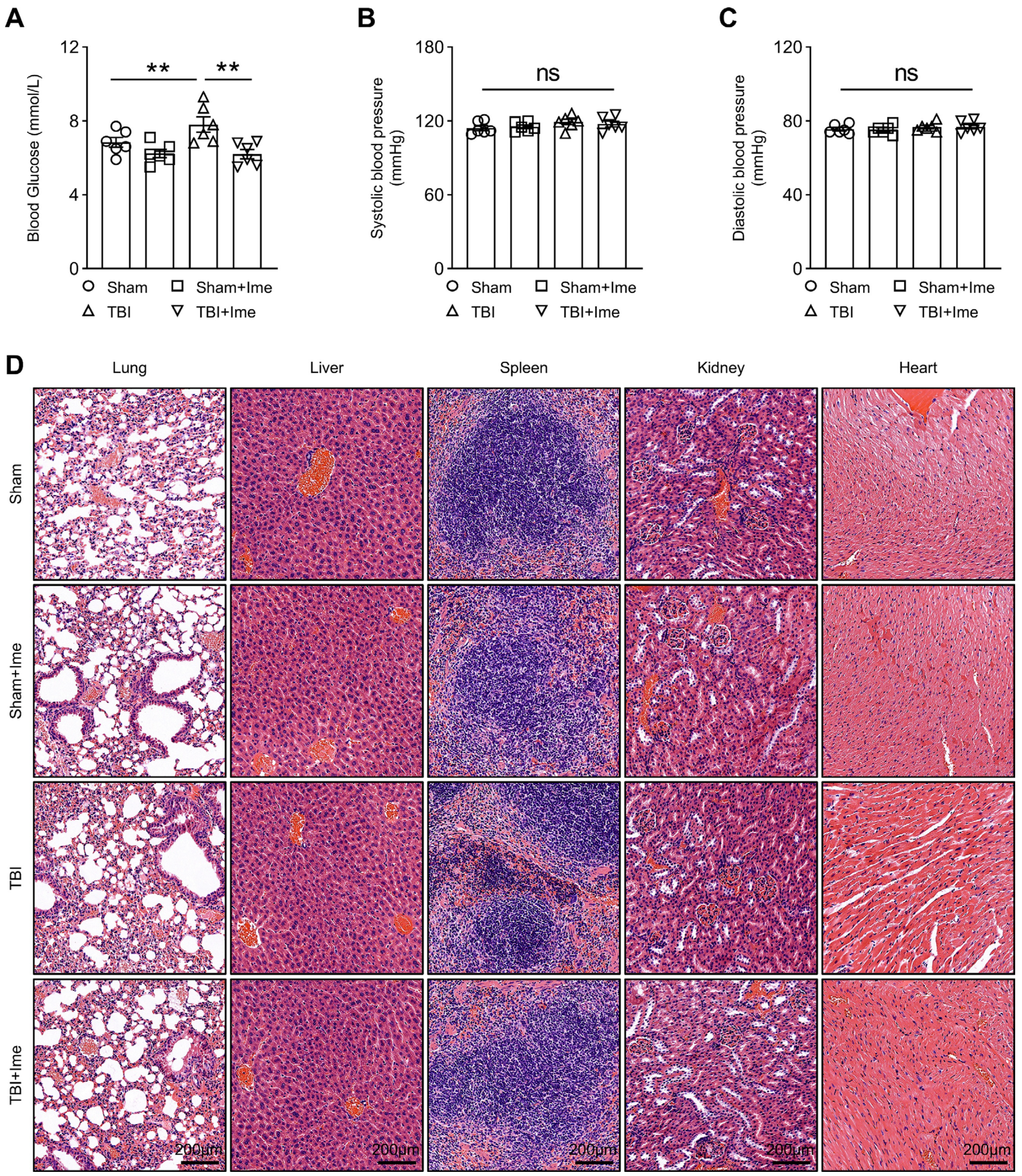

Supplemental Figure 17. Imeglimin treatment has a safety profile. (A) Bar plot shows levels of blood glucose from Sham, Sham+Ime, TBI, and TBI+Ime mice. n=6/group. **(B-C)** Bar plots show systolic and diastolic blood pressure of Sham, Sham+Ime, TBI, and TBI+Ime mice. n=6/group. **(D)** Representative histological section results of lung, liver, spleen, kidney and heart under different conditions of Imeglimin treatment. Data are represented as mean ± SEM. $**P < 0.01$. Statistical analyses were performed using two-way ANOVA followed by Tukey post hoc test.

Supplemental Table 1. Summary Clinical Information for TBI Patients.

	**Young TBI**	**Aged TBI**
Cohort size	13	22
Age (years)(min-max)	19-59	65-86
Sex (male), n (%)	9 (69.23%)	15 (68.18%)
Pupil abnormalities, n (%)	8 (61.54%)	10 (45.45%)
Polytrauma, n (%)	3 (23.08%)	3 (13.64%)
Moderate TBI (GCS 8–12), n (%)	7 (53.85%)	14 (63.64%)
Severe TBI (GCS 3–7), n (%)	6 (46.15%)	8 (36.36%)
In hospital mortality, n (%)	2 (15.38%)	4 (18.18%)
Length of hospitalization (days), Median (IQR)	12.3 (10,21)	17.9 (12,23)
GCS at discharge (survivors), Median (IQR)	11.6 (7,15)	10.4 (6,15)

Supplemental Table 2. Detailed Clinical Information for TBI Patients.

Patient Number	Sex	Age	Causes of TBI	Location of the injured area	Type of injury
1	Male	30	Traffic accident	Right frontotemporal	Cerebral contusion with subdural
2	Female	26	Traffic accident	Right frontotemporal	Cerebral contusion hematoma and
3	Male	19	Traffic accident	Right temporal	Cerebral contusion with epidural
4	Male	21	Traffic accident	Left frontotemporal	Cerebral contusion hematoma
5	Male	29	Traffic accident	Bilateral frontotemporal	Cerebral contusion with subdural
6	Male	31	Traffic accident	Right parietal	Cerebral contusion hematoma and
7	Female	33	Traffic accident	left frontal	Cerebral contusion hematoma
8	Male	38	Traffic accident	Right frontotemporal	Isolated epidural hematoma

9	Female	34	Traffic accident	Left frontotemporal	Cerebral contusion with subdural
10	Male	35	Fall	Left frontotemporal	Cerebral contusion with subdural
11	Male	45	Traffic accident	Right frontotemporal	Cerebral contusion hematoma
12	Male	59	Traffic accident	Bilateral frontotemporal	Cerebral contusion hematoma and
13	Female	23	Traffic accident	Bilateral frontotemporal	Cerebral contusion with subdural
14	Female	76	Fall	Right frontal	Cerebral contusion hematoma
15	Female	67	Traffic accident	Left temporal	Ccerebral contusion hematoma
16	Male	66	Traffic accident	Left temporo-occipital	Cerebral contusion hematoma
17	Female	69	Traffic accident	Left temporal	Cerebral contusion with subdural

18	Female	73	Traffic accident	Left frontotemporal	Cerebral contusion hematoma
19	Male	79	Fall	Left frontal, right occipital	Cerebral contusion with subdural
20	Male	81	Fall	Left frontotemporal	Cerebral contusion with subdural
21	Male	83	Fall	left frontal	Cerebral contusion with subdural
22	Male	62	Traffic accident	Right frontotemporal	Cerebral contusion with subdural
23	Male	62	Traffic accident	Left frontotemporal	Cerebral contusion hematoma
24	Male	86	Fall	Left frontotemporal	Cerebral contusion with subdural
25	Male	63	Traffic accident	Left temporal	Cerebral contusion with subdural
26	Male	65	Traffic accident	Left temporal	Cerebral contusion hematoma

27	Male	66	Traffic accident	Right frontotemporal	Cerebral contusion with subdural
28	Male	71	Traffic accident	Right temporal	Cerebral contusion with subdural
29	Male	66	Traffic accident	Left frontotemporal	Cerebral contusion hematoma
30	Male	67	Traffic accident	Right frontal	Cerebral contusion hematoma and
31	Female	70	Traffic accident	Left frontal	Cerebral contusion hematoma
32	Female	71	Traffic accident	Right frontotemporal	Cerebral contusion hematoma
33	Male	72	Traffic accident	Right frontotemporal	Cerebral contusion with subdural
34	Male	75	Traffic accident	Left frontal	Cerebral contusion hematoma
35	Female	76	Traffic accident	Left temporal	Cerebral contusion hematoma

Supplemental Table 3. Antibodies used in this work.

Antibodies	Source	Application	Dilution
CD45 Monoclonal Antibody (HI30), PerCP-Cyanine5.5, eBioscience™	Invitrogen	FC	1:1000
CD11b Monoclonal Antibody (ICRF44), Alexa Fluor™ 700, eBioscience™	Invitrogen	FC	1:1000
Lysozyme Monoclonal Antibody (LZ-2), FITC	Invitrogen	FC	1:1000
Human/Mouse NLRP3/NALP3 APC-conjugated Antibody	R＆D systems	FC	1:1000
CD45 Monoclonal Antibody (30-F11), PerCP-Cyanine5.5, eBioscience™	Invitrogen	FC	1:1000
CD11b Monoclonal Antibody (M1/70), Super Bright™ 702, eBioscience™	Invitrogen	FC	1:1000
Tmem119 Monoclonal Antibody (V3RT1GOsz), PE, eBioscience™	Invitrogen	FC	1:1000
Lysozyme Monoclonal antibody	Proteintech	FC	1:1000
Monoclonal Anti-TMEM119 antibody	Sigma-Aldrich	IF/FC	1:500
NLRP3 Monoclonal Antibody (768319)	Invitrogen	IF	1:200

Lysozyme Polyclonal Antibody	Invitrogen	IF	1:200
GFAP Polyclonal antibody	Proteintech	IF	1:200
NeuN Polyclonal antibody	Proteintech	IF	1:200
TMEM119 Polyclonal antibody	Proteintech	IF	1:200
FlexAble 2.0 CoraLite® Plus 594 Antibody Labeling Kit for Mouse IgG1	Proteintech	FC	1:100
FlexAble 2.0 CoraLite® Plus 488 Antibody Labeling Kit for Mouse IgG1	Proteintech	FC	1:100
Donkey Anti-Rat IgG H&L (Alexa Fluor® 488) (ab150153)	Abcam	IF	1:1000
Donkey Anti-Rabbit IgG H&L (Alexa Fluor® 488) (ab150073)	Abcam	IF	1:1000
Donkey Anti-Mouse IgG H&L (Alexa Fluor® 594) (ab150108)	Abcam	IF	1:1000

Supplemental Table 4. PCR sequences.

Gene	Forward primer sequence (5’-3’)	Reverse primer sequence (5’-3’)
IL1B (hu)	CCACAGACCTTCCAGGAGAATG	GTGCAGTTCAGTGATCGTACAGG
IL6 (hu)	AGACAGCCACTCACCTCTTCAG	TTCTGCCAGTGCCTCTTTGCTG
TNF (hu)	CTCTTCTGCCTGCTGCACTTTG	ATGGGCTACAGGCTTGTCACTC
IL4 (hu)	CCGTAACAGACATCTTTGCTGCC	GAGTGTCCTTCTCATGGTGGCT
IL10 (hu)	TCTCCGAGATGCCTTCAGCAGA	TCAGACAAGGCTTGGCAACCCA
TGFB1 (hu)	TACCTGAACCCGTGTTGCTCTC	GTTGCTGAGGTATCGCCAGGAA
Il1b (ms)	TGGACCTTCCAGGATGAGGACA	GTTCATCTCGGAGCCTGTAGTG
Il6 (ms)	TACCACTTCACAAGTCGGAGGC	CTGCAAGTGCATCATCGTTGTTC
Tnf (ms)	GGTGCCTATGTCTCAGCCTCTT	GCCATAGAACTGATGAGAGGGAG
Il4 (ms)	ATCATCGGCATTTTGAACGAGGTC	ACCTTGGAAGCCCTACAGACGA
Il10 (ms)	CGGGAAGACAATAACTGCACCC	CGGTTAGCAGTATGTTGTCCAGC
Il18 (ms)	GACAGCCTGTGTTCGAGGATATG	TGTTCTTACAGGAGAGGGTAGAC
Cdkn1a (ms)	TCGCTGTCTTGCACTCTGGTGT	CCAATCTGCGCTTGGAGTGATAG
Cdkn2a (ms)	TGTTGAGGCTAGAGAGGATCTTG	CGAATCTGCACCGTAGTTGAGC
Tgfb1 (ms)	TGATACGCCTGAGTGGCTGTCT	CACAAGAGCAGTGAGCGCTGAA
Elf1 (ms)	ACCCAGCTCTTCCGAACTGTTC	AGGAGACACCACTACTGGAACC
Nfkb2 (ms)	TGCTGATGGCACAGGACGAGAA	GTTGATGACGCCGAGGTACTGA
Rel (ms)	GAAGACTGCGACCTCAATGTGG	TCTTGTTCACACGGCAGATCCTT
Cebpb (ms)	CAACCTGGAGACGCAGCACAAG	GCTTGAACAAGTTCCGCAGGGT
Rela (ms)	TCCTGTTCGAGTCTCCATGCAG	GGTCTCATAGGTCCTTTTGCGC
Fli1 (ms)	CCATACAGACCAGTCCTCACGA	CATGGTCTGTGATCCTCCAAGG
Nfkb1 (ms)	GCTGCCAAAGAAGGACACGACA	GGCAGGCTATTGCTCATCACAG

Stat3 (ms)	AGGAGTCTAACAACGGCAGCCT	GTGGTACACCTCAGTCTCGAAG
Jun (ms)	CAGTCCAGCAATGGGCACATCA	GGAAGCGTGTTCTGGCTATGCA
Junb (ms)	GACCTGCACAAGATGAACCACG	ACTGCTGAGGTTGGTGTAGACG
Fosb (ms)	ACCTGTCTTCGGTGGACTCCTT	TGGCTGGTTGTGATTGCGGTGA
Fos (ms)	GGGAATGGTGAAGACCGTGTCA	GCAGCCATCTTATTCCGTTCCC
Relb (ms)	GTTCTTGGACCACTTCCTGCCT	TAGGCAAAGCCATCGTCCAGGA
Jund (ms)	ACCTGCACAAGCAAAGCCAGCT	CGAAACTGCTCAGGTTGGCGTA
Mef2c (ms)	GTGGTTTCCGTAGCAACTCCTAC	GGCAGTGTTGAAGCCAGACAGA
Atf4 (ms)	AACCTCATGGGTTCTCCAGCGA	CTCCAACATCCAATCTGTCCCG
Batf (ms)	CACAGAAAGCCGACACCCTTCA	GCTGCTCAGCACTGATGTGAAG
Atf3 (ms)	GAAGATGAGAGGAAAAGGAGGCG	GCTCAGCATTCACACTCTCCAG
Nfe2l2 (ms)	CAGCATAGAGCAGGACATGGAG	GAACAGCGGTAGTATCAGCCAG
Maff (ms)	ACCTGTCGGATGAAGCGCTGAT	TAGCCGCGGTTCTTGAGTGTGC
Tgif1 (ms)	CAGATTCTGCGAGACTGGCTGT	CGGGCGTTGATGAACCAGTTAC
Zbtb7a (ms)	TGCGAGAAGGTGATTCAGGGTG	TTCCGCATGTGCACCTTCAGCT
Nr3c1 (ms)	TGGAGAGGACAACCTGACTTCC	ACGGAGGAGAACTCACATCTGG
Bach1 (ms)	CCATGACATCCGCAGAAGGAGT	GCGTTGACAGAATGTGGTCTCG
Bcl3 (ms)	AGCAGTCGTCTCAGCTCCAATG	AGGCAGGTGTAGATGTTGTGGG
Cebpa (ms)	GCAAAGCCAAGAAGTCGGTGGA	CCTTCTGTTGCGTCTCCACGTT
Egr1 (ms)	AGCGAACAACCCTATGAGCACC	ATGGGAGGCAACCGAGTCGTTT
Ets2 (ms)	GTGGCTTCCAAAAGGAGCAACG	TTCACCAGGCTGAACTCGTTGG
Xbp1 (ms)	TGGACTCTGACACTGTTGCCTC	TAGACCTCTGGGAGTTCCTCCA
Ccl3 (ms)	ACTGCCTGCTGCTTCTCCTACA	ATGACACCTGGCTGGGAGCAAA
Ccl4 (ms)	ACCCTCCCACTTCCTGCTGTTT	CTGTCTGCCTCTTTTGGTCAGG

Ccl5 (ms)	CCTGCTGCTTTGCCTACCTCTC	ACACACTTGGCGGTTCCTTCGA
Ccr5 (ms)	GTCTACTTTCTCTTCTGGACTCC	CCAAGAGTCTCTGTTGCCTGCA
Ccr6 (ms)	ACAGAGCCATCCGAGTCGTGAT	CTGGTGTAGGCGAGGACTTTCT
Actb (ms)	CATTGCTGACAGGATGCAGAAGG	TGCTGGAAGGTGGACAGTGAGG
ELF1 (hu)	CTAAAGCAGTGTCCAGGTTGTGG	CGCTGACCTTCCACTTTTGCCA
NFKB2 (hu)	GGCAGACCAGTGTCATTGAGCA	CAGCAGAAAGCTCACCACACTC
REL (hu)	AGTTGCGGAGACCTTCTGACCA	CGTGATCCTGGCACAGTTTCTG
CEBPB (hu)	AGAAGACCGTGGACAAGCACAG	CTCCAGGACCTTGTGCTGCGT
RELA (hu)	TGAACCGAAACTCTGGCAGCTG	CATCAGCTTGCGAAAAGGAGCC
FLI1 (hu)	ACGGAAGTGCTGTTGTCACACC	CAAGCTCCTCTTCTGACTGAGTC
NFKB1 (hu)	GCAGCACTACTTCTTGACCACC	TCTGCTCCTGAGCATTGACGTC
STAT3 (hu)	CTTTGAGACCGAGGTGTATCACC	GGTCAGCATGTTGTACCACAGG
JUN (hu)	CCTTGAAAGCTCAGAACTCGGAG	TGCTGCGTTAGCATGAGTTGGC
JUNB (hu)	CGATCTGCACAAGATGAACCACG	CTGCTGAGGTTGGTGTAAACGG
FOSB (hu)	TCTGTCTTCGGTGGACTCCTTC	GTTGCACAAGCCACTGGAGGTC
FOS (hu)	GCCTCTCTTACTACCACTCACC	AGATGGCAGTGACCGTGGGAAT
RELB (hu)	TGTGGTGAGGATCTGCTTCCAG	TCGGCAAATCCGCAGCTCTGAT
JUND (hu)	ATCGACATGGACACGCAGGAGC	CTCCGTGTTCTGACTCTTGAGG
MEF2C (hu)	TCCACCAGGCAGCAAGAATACG	GGAGTTGCTACGGAAACCACTG
ATF4 (hu)	TTCTCCAGCGACAAGGCTAAGG	CTCCAACATCCAATCTGTCCCG
BATF (hu)	GATGTGAGAAGAGTTCAGAGGAG	GTTTCTCCAGGTCTTCGCTCTC
ATF3 (hu)	CGCTGGAATCAGTCACTGTCAG	CTTGTTTCGGCACTTTGCAGCTG
NFE2L2 (hu)	CACATCCAGTCAGAAACCAGTGG	GGAATGTCTGCGCCAAAAGCTG
MAFF (hu)	CTGTCGGACGAGGCGCTGATG	AGCCACGGTTTTTGAGTGTGCG

TGIF1 (hu)	GGATTGGCTGTATGAGCACCGT	GCCATCCTTTCTCAGCATGTCAG
ZBTB7A (hu)	GCAACATCTGCAAGGTCCGCTT	TCTTCAGGTCGTAGTTGTGGGC
NR3C1 (hu)	GGAATAGGTGCCAAGGATCTGG	GCTTACATCTGGTCTCATGCTGG
BACH1 (hu)	CACCGAAGGAGACAGTGAATCC	GCTGTTCTGGAGTAAGCTTGTGC
BCL3 (hu)	GAACACCGAGTGCCAAGAAACC	GCTAAGGCTGTTGTTTTCCACGG
CEBPA (hu)	AGGAGGATGAAGCCAAGCAGCT	AGTGCGCGATCTGGAACTGCAG
EGR1 (hu)	AGCAGCACCTTCAACCCTCAGG	GAGTGGTTTGGCTGGGGTAACT
ETS2 (hu)	ACTCCGCCAACTGTGAATTGCC	CCACTGGCATACCTGTTGCTCA
XBP1 (hu)	CTGCCAGAGATCGAAAGAAGGC	CTCCTGGTTCTCAACTACAAGGC
ACTB (hu)	CACCATTGGCAATGAGCGGTTC	AGGTCTTTGCGGATGTCCACGT

Supplemental Table 5. Modified Neurological Severity Score points.

Motor tests	6
Raising mice by tail	3
Flexion of forelimb	1
Flexion of hindlimb	1
Head moved >10° to vertical axis within 30 s	1
Placing mice on floor (normal=0; maximum=3)	3
Normal walk	0
Inability to walk straight	1
Circling toward paretic side	2
Falls down to paretic side	3
Sensory tests	2
Placing test (visual and tactile test)	1
Proprioceptive test (deep sensation, pushing paw against table edge to stimulate limb muscles)	1
Beam balance tests (normal=0; maximum=6)	6
Balances with steady posture	0
Grasps side of beam	1
Hugs beam and 1 limb falls down from beam	2
Hugs beam and 2 limbs fall down from beam, or spins on beam (>60 s)	3
Attempts to balance on beam but falls off (>40 s)	4

Attempts to balance on beam but falls off (>20 s)	5
Falls off; no attempt to balance or hang on to beam (<20 s)	6
Reflex absence and abnormal movements	4
Pinna reflex (head shake when auditory meatus is touched)	1
Corneal reflex (eye blink when cornea is lightly touched with cotton)	1
Startle reflex (motor response to a brief noise from snapping a clipboard paper)	1
Seizures, myoclonus, myodystony	1
Maximum points	18

Supplemental Table 6. sgRNA sequences.

Gene	Sg sequence (5'-3')	ICE indel(%)
ITGAM Sg	ATCCTAGTTGTCATCACGGA	74
CEBPB Sg	GGCCAACTTCTACTACGAGG	79
ELF1 Sg1	ACATGTTCCACAATTACGGC	71
ELF1 Sg2	ATTTGCTAGTAACGTCATGG	54
ELF1 Sg3	ATGTGTCCGTCACATTAGAT	50
FLI1 Sg	CGCTTGACGTTGACCCTCAC	69
FOS Sg	GGCGTTGTGAAGACCATGAC	53
FOSB Sg	CCACCAGCGGAACTACCAGT	71
ITGAM Sg	ATCCTAGTTGTCATCACGGA	74
JUN Sg	TGATAATCCAGTCCAGCAAC	58
JUNB Sg	CGACGACTCATACACAGCTA	54
NFKB1 Sg	CAACTATGTGGGACCAGCAA	85
NFKB2 Sg	TAGGCTGTTCCACGATCACC	79
REL Sg	CACATCGAATACCCAAATTT	67
RELA Sg	AGCTGATGTGCACCGACAAG	59
STAT3 Sg	ACAATCCGGGCAATCTCCAT	84

Supplemental Table 7. Canonical marker genes used to identify microglia and macrophage.

Cell type	Canonical marker genes
Microglia	*P2ry12*, *Tmem119*, *Cx3cr1*, *Csf1r*, *Sall1*
Macrophage	*Lyve1*, *Pf4*, *Mrc1*, *Ms4a3*
