# Supplementary figures and images for: Aging-dependent microglial heterogeneity worsens outcomes in models of traumatic brain injury
Source: J Clin Invest. 2026 Apr 2;136(12):e196112. doi: 10.1172/JCI196112 (PMC13262727; doi:10.1172/JCI196112)

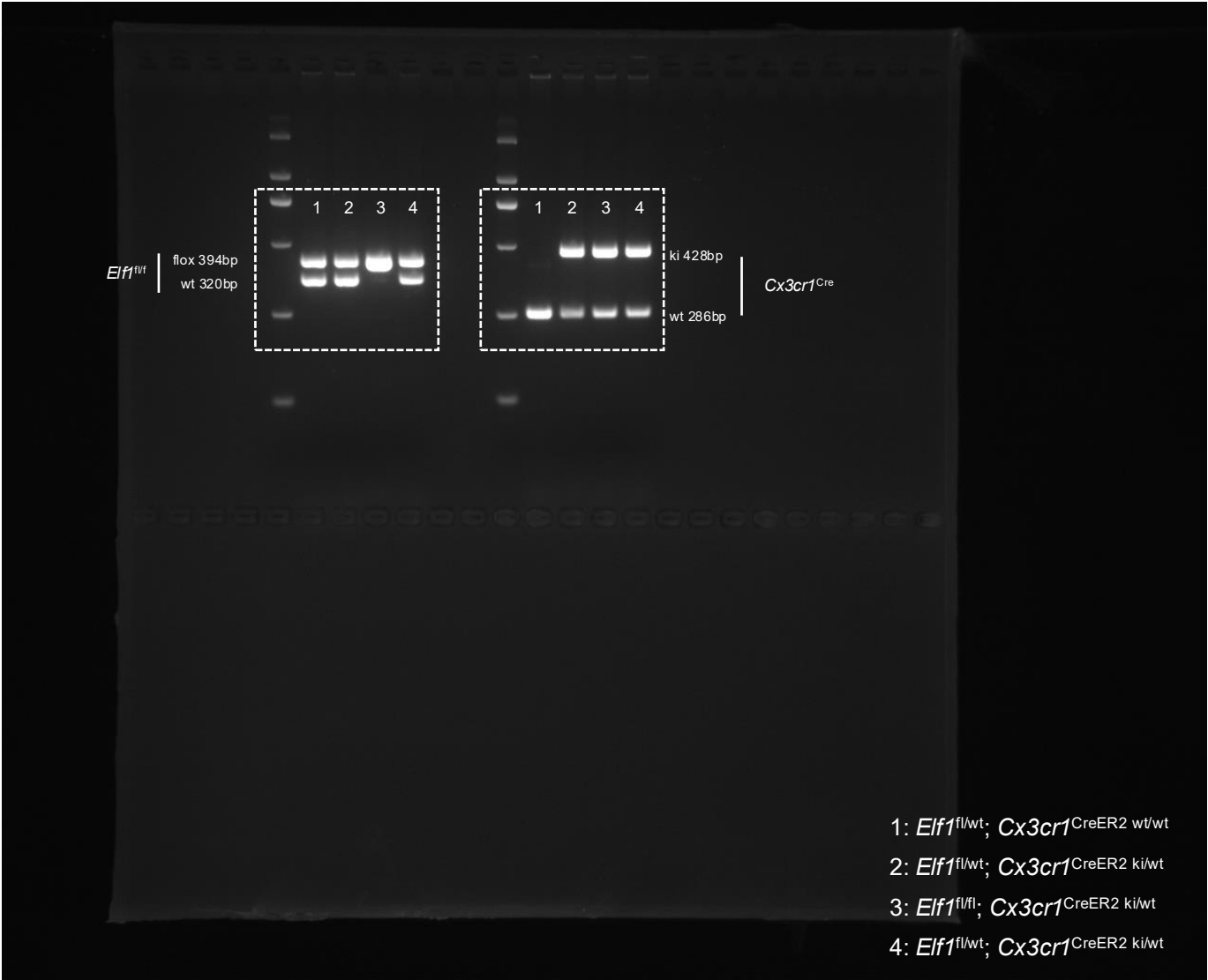

Supplement: Unedited blot and gel images [file jci-136-196112-s297.pdf]
